# A reaction-time adjusted PSI method for estimating performance in the stop-signal task

**DOI:** 10.1371/journal.pone.0210065

**Published:** 2018-12-31

**Authors:** Lorenz Weise, Maren Boecker, Siegfried Gauggel, Bjoern Falkenburger, Barbara Drueke

**Affiliations:** 1 Department of Medical Psychology and Medical Sociology, RWTH Aachen University, Aachen, Germany; 2 Department for Neurology, RWTH Aachen University, Aachen, Germany; 3 JARA-BRAIN Institute for Molecular Neuroscience and Neuroimaging, Forschungszentrum Jülich GmbH, RWTH Aachen University, Aachen, Germany; University of Wuerzburg, GERMANY

## Abstract

A central experimental task in executive control research is the Stop-signal task, which allows measuring the ability to inhibit dominant responses. A crucial aspect of this task consists of varying the delay between the Go- and Stop-signal. Since the time necessary to administer the task can be long, a method of optimal delay choice was recently proposed: the PSI method. In a behavioral experiment, we show a variant of this method, the PSI marginal method, to be unable to deal with the Go-response slowing often observed in the Stop-signal task. We propose the PSI adjusted method, which is able to deal with this response slowing by correcting the estimation process for the current reaction time. In several sets of behavioral simulations, as well as another behavioral experiment, we document and compare the statistical properties of the PSI marginal method, our PSI adjusted method, and the traditional staircase method, both when reaction times are constant and when they are linearly increasing. The results show the PSI adjusted method’s performance to be comparable to the PSI marginal method in the case of constant Go-response times, and to outperform the PSI marginal method as well as the staircase methods when there is response slowing. The PSI adjusted method thus offers the possibility of efficient estimation of Stop-signal reaction times in the face of response slowing.

## Introduction

Among executive functions, the ability to inhibit and change actions in order to regulate impulses and attain long-term goals has a central place. A core experimental method used to measure the concept of response inhibition is the Stop-signal task, originally developed by Logan and Cowan [1]. In this task, on a majority of trials (Go-trials) a Go-signal is presented, to which participants have to respond as quickly as possible, producing the Go-reaction time (Go-RT). On a minority of trials (Stop-trials), the Go-signal is followed at some delay (the Stop-signal delay, SSD) by a Stop-signal, which instructs the participant to withhold the Go-response. A fundamental result is that withholding the Go-response becomes more difficult with longer SSDs [1].

An influential model of the Stopping-process in the Stop-signal task is the independent horse-race model [1]. The horse-race model assumes that there are two independent processes at play during a Stop-trial: the Go-process, which starts with presentation of the Go-signal and concludes with a Go-response, and the Stop-process, starting with the presentation of the Stop-signal and concluding with the withholding of the Go-response. Both processes race to conclusion and whichever finishes first determines the response. Since a longer delay between Go- and Stop-signal gives the Stop-process less time to finish, trials with longer SSDs make it harder to correctly withhold the Go-response. In a common Stop-signal experiment, a participant will manage to withhold the Go-response only on some Stop-trials, but not on others, producing an erroneous response. The Go- and Stop-process are thought to be largely independent (for a review, see [2]).

The horse-race model allows computation of the time the stopping process takes (the Stop-signal reaction time, SSRT). The SSRT can be computed by determining the SSD at which the likelihood of producing an erroneous Go-response (referred to as *p(error)*) is 0.5, and then subtracting that SSD from the mean Go-RT during simple Go-trials. The SSD for which *p(error)* is 0.5 is referred to as the critical SSD. To estimate the critical SSD, the staircase tracking procedure is often used. With this method, a correctly withheld Go-response increases the SSD by a certain duration, while an erroneously executed Go-response decreases the SSD by a certain amount. In practice, this method usually converges on an SSD at which *p(error)* is at 0.5, such that this SSD can be subtracted from the mean Go-RT (this form of computing the SSRT is known as the *mean method*). Should the method converge at a *p* different from 0.5, the Go-RTs are rank-ordered, and the SSD is subtracted from the *n*th Go-RT, where *n* is the total number of Go-RTs multiplied by *p* (the so-called *integration method*, [2]). An illustration of the horse race model’s different quantities can be found in the supplementary material (S1 Fig).

One disadvantage of the Stop-signal task is its potential length, especially if different conditions are included, because for every Stop-trial several Go-trials have to be included to make the Go-response the prepotent response, and because it takes a relatively large number of Stop-trials to reliably estimate the SSRT [3]. As a possible solution to this problem, Livesey and Livesey [4] proposed a different method to select an upcoming Stop-trial’s SSD and estimate the critical SSD used for computing the SSRT, based on an adaptive Bayesian estimation algorithm known in the psychophysical literature as the PSI method [5].

In the following, our description of the method closely follows the original work of Kontsevich and Tyler [5], which should be consulted for further detail. We present the full algorithm here so that changes in its application to the Stop-signal task discussed later in this article will be easier to follow.

In short, the relationship between SSD and the likelihood of a response in a Stop-trial follows a psychometric function, such as the Weibull cumulative density function chosen by Livesey and Livesey [4]:

$$p(\mathrm{success}|\mathrm{SSD},\mathrm{Threshold},\mathrm{Slope},\mathrm{ErrorRate})=(1-2*\mathrm{ErrorRate})+\left(1-2^{{-(\frac{\mathrm{SSD}}{\mathrm{Threshold}})}^{\mathrm{Slope}}}\right)+\mathrm{ErrorRate}$$ (1)

p(error|SSD,Threshold,Slope,ErrorRate)=1−p(success|SSD,Threshold,Slope,ErrorRate) (2)

The *Threshold* parameter governs at which SSD *p(error)* is 0.5, the *Slope* determines the rise of the function, and the *ErrorRate* determines the minimum and maximum the function can take on.

A participant’s performance in the task is described by a vector *λ*_*participant*_ = (*Threshold*_*participant*_, *Slope*_*participant*_, *ErrorRate*_*participant*_) in this three-dimensional parameter space. Our knowledge of a participant’s performance *λ*_*participant*_ at the start of the task is formalized as a probability distribution p_0_(λ) over the parameter space, which assigns each possible performance vector λ a probability p_0_ of matching the participant’s performance *λ*_*participant*_. When having no prior knowledge, at the outset of the task we can set p_0_(λ) as the uniform distribution.

Step 1: At the outset of Stop-trial 1, p_0_ represents our prior knowledge of the participant’s performance. For each SSD and response that is possible in trial 1, we can compute the posterior probability of each parameter vector λ, according to Bayes’ theorem:

$$p_{0}(\lambda |\mathrm{SSD},\mathrm{response})=\frac{p_{0}(\lambda )*p(\mathrm{response}|\lambda ,\mathrm{SSD})}{\sum_{\lambda }p_{0}(\lambda )*p(\mathrm{response}|\lambda ,\mathrm{SSD})}$$ (3)

where *p*(*response*|*λ*,*SSD*) is the psychometric function in Eqs 1 and 2. Thus, for each SSD we might present in the upcoming trial 1, and for each response the participant might give in return, we compute the posterior distribution over the parameter space.

Step 2: For each of these possible SSD-response combinations we compute the entropy *H*_0_ of the resulting posterior distribution *p*_0_(*λ*|*SSD*,*response*) as:

$$H_{0}(\mathrm{SSD},\mathrm{response})=-\sum_{\lambda }p_{0}(\lambda |\mathrm{SSD},\mathrm{response})*log\left(p_{0}(\lambda |\mathrm{SSD},\mathrm{response})\right)$$ (4)

The entropy function stems from information theory and quantifies the amount of information necessary to fully specify the state of a system [6]. In the current context, the entropy of each posterior distribution quantifies how much information is still needed to completely specify which vector *λ* in parameter space corresponds to our participant’s performance vector *λ*_*participant*_. When deciding which SSD to offer on Stop-trial 1, we choose that SSD which yields the lowest entropy, and thus the most information about the participant’s performance.

Step 3: Since the entropy function in Eq 4 is also dependent on the participant’s response, we compute the current likelihood of the two possible responses (success, error) for each SSD, using the prior probability distribution p_0_(λ) and the psychometric functions in Eqs 1 and 2:

$$p_{0}(\mathrm{success}|\mathrm{SSD})=\sum_{\lambda }p(\mathrm{success}|\lambda ,\mathrm{SSD})*p_{0}(\lambda )$$ (5)

$$p_{0}(\mathrm{error}|\mathrm{SSD})=\sum_{\lambda }p(\mathrm{error}|\lambda ,\mathrm{SSD})*p_{0}(\lambda )$$ (6)

With these, we can compute the expected entropy *E*[*H*_0_(*SSD*)] as

$$E[H_{0}(\mathrm{SSD})]=H_{0}(\mathrm{SSD},\mathrm{success})*p_{0}(\mathrm{success}|\mathrm{SSD})+H_{0}(\mathrm{SSD},\mathrm{error})*p_{0}(\mathrm{error}|\mathrm{SSD})$$ (7)

Step 4: In Stop-trial 1, we present the SSD yielding the lowest expected entropy and record the participant’s response.

Step 5: After having finished trial 1, our updated prior knowledge *p*_1(λ)_ is the posterior distribution *p*_0_(*λ*|*SSD*,*response*) from Eq 3 corresponding to the SSD we eventually offered in trial 1 and the response the participant gave. We now continue with the following trials *t* = 2, 3 … *n*, and on each trial, in order to determine the SSD to offer, steps 1 to 4 are now repeated, except that where we used our previous prior distribution *p*_0_(*λ*), we now use our updated prior distribution *p*_*t*−1(λ)_.

Step 6: At the end of the task, after *n* Stop-trials have been concluded, we derive the parameter vector most likely to match our participant’s parameter vector *λ*_*participant*_ by computing the expected value of *λ*:

$$E[\lambda ]=\sum_{\lambda }\lambda *p_{n}(\lambda )$$ (8)

In a number of Stop-signal task simulations and behavioral experiments, Livesey and Livesey [4] showed that the PSI method can quickly and accurately estimate the SSRT. In terms of correlation with the actually simulated SSRT, as well as mean absolute deviation, the PSI method was comparable to the traditional staircase. However, the staircase was shown to provide a systematically biased estimate of the SSRT, overestimating short SSRTs and underestimating long SSRTs, even at more than 30 Stop-trials, while the PSI method arrived at a relatively unbiased estimate already after 10 Stop-trials. The PSI method thus promises as a viable alternative to the staircase method when quick and unbiased estimation of the SSRT is required.

The purpose of this study is to investigate the influence of Go-response slowing on the PSI method. In Livesey and Livesey [4], the Go-RTs in both the simulations and the behavioral tasks are constant over the course of the experiment. However, it is known that the Stop-signal task can invite a gradual slowing of the Go-RTs over the course of the experiment [7, 8]. There is a strategic element to this slowing, since participants will sometimes try to slow their Go-responses in order to still catch the Stop-signal. Since this increases the participant’s chance of correctly responding, the staircase algorithm will respond to this by further increasing the SSD, motivating the participant to further slow the Go-response. In this way, the staircase even promotes this strategic slowing. It is an open question how the PSI method can deal with this slowing effect in the Stop-signal task. In order to test this, we employ a version of the so-called Stop-change task, a variant of the Stop-signal task we have previously found to elicit strong Go-response slowing. Previous research has shown the processes involved in stopping and changing to be partly overlapping [9–12].

In the following behavioral study we implement a slightly modified version of the PSI method, the so-called PSI marginal method [13, 14]. In the original PSI method, as used by Livesey and Livesey [4], the error rate determining the minimum and maximum *p(error)* the psychometric function can take on is usually fixed. Prins [13] showed that this can lead to a biased estimation of the threshold parameter, which is central to estimating the SSRT in the Stop-signal task. As a remedy, Prins proposed also adding the error rate-parameter to the parameter space, thus estimating three free parameters instead of two. As a further refinement, he proposed minimizing the entropy of a posterior distribution that is marginalized over irrelevant parameters (e.g. in the Stop-signal task, minimizing entropy of a posterior that includes the threshold parameter but is marginalized over slope and error rate, since these are not relevant to SSRT estimation).

In terms of the formulation above, this means changing Eq 4 so as not to compute the entropy *H*_0_(*SSD*,*response*) of the full posteriors, but instead to compute:

$$H_{0‑\mathrm{marginal}}(\mathrm{SSD},\mathrm{response})=-\sum_{\lambda }p_{0}(\mathrm{Threshold}|\mathrm{SSD},\mathrm{response})*log\left(p_{0}(\mathrm{Threshold}|\mathrm{SSD},\mathrm{response})\right)$$ (9)

where *p*_0_(*Threshold*|*SSD*,*response*) is the marginalized posterior distribution:

$$p_{0}(\mathrm{Threshold}=t|\mathrm{SSD},\mathrm{response})=\sum_{s}\sum_{e}p_{0}(\mathrm{Threshold}=t,\mathrm{Slope}=s,\mathrm{ErrorRate}=e|\mathrm{SSD},\mathrm{response})$$ (10)

Since we implement these recommendations, we will refer to the method we tested as the PSI marginal method from now on, and will refer to the method proposed by Livesey and Livesey as the original PSI method.

## Behavioral experiment 1: the PSI marginal method

### Methods

#### Participants

Participants were 24 students or former students (M_age_ = 23.8 ± 2.9, 14 male, 1 left-handed) with normal or corrected-to-normal vision and no history of neurological or mental illness. Participants were paid 15 € for their participation and provided written informed consent after receiving information about the general aims and risks associated with participation as well as their right to withdraw consent at any time. The experiment was approved by the ethics committee of the university hospital of the RWTH Aachen and conducted in accordance with the Declaration of Helsinki.

#### Task

Participants performed a Stop-change task, in which they had to not only inhibit a response but also change it to another one. The Go-stimuli were two filled green squares, one on the left and one on the right side of the screen, to which participants responded by pressing the ‘f’- and ‘j’-keys on the keyboard with left and right index fingers simultaneously and as quickly as possible. On Change-trials, one of the green squares turned red, indicating that on this side, the index finger response should be inhibited and replaced by a middle finger response to the ‘d’- or ‘k’-key.

Before each Go-signal, a cue appeared that could give different degrees of information about the upcoming trial. The cue consisted of two hollow squares. In the foreknowledge condition, the cue would indicate on which side the Change-signal could appear (i.e. a green hollow square left and a red hollow square right, indicating a Change-signal might appear on the right). The cue was not completely informative, since the upcoming trial might still be a Go-trial, but if a Change-signal appeared, it was always on the cued side. In the no-foreknowledge condition, both hollow squares were red, such that on both sides a Change-signal might appear. The factor foreknowledge [yes, no] was crossed with the factor side [left, right], which corresponds to the side on which the Change-signal appeared. Consequently, there were four experimental conditions: foreknowledge-left; foreknowledge-right; no-foreknowledge-left; no-foreknowledge-right. In addition to these Change-related conditions, another condition was included (“certain Go”), which did not include Change-trials. In this condition, the foreknowledge cue consisted of two hollow green squares, indicating that no Change-signal was going to appear.

While these different conditions are usually used to gauge the influence of foreknowledge and certainty of the stopping process, they are not relevant to our further discussion of the task, which we mainly used because it evokes substantial response slowing. However, a summary table of behavioral performance of the different conditions can be found in the supplementary materials (S1 and S2 Tables). The foreknowledge cue remained on the screen for 1500 ms, followed by a 500 ms fixation cross, followed by the Go-stimulus. The Go-stimulus ended with the response or after 2500 ms, followed by an 850 ms inter trial interval. The experiment was performed using Presentation software (Version 18.0, Neurobehavioral Systems, Inc., Berkeley, CA, www.neurobs.com).

#### Procedure

The experiment started with a training run, introducing participants to the different conditions of the Stop-change task. Subsequently, participants completed two runs of the full task, one with the SSD controlled by the traditional staircase tracking procedure, the other controlled by the PSI marginal algorithm. The staircase tracking procedure used a step-size of 50 ms, starting at 250 ms and being limited to 50 ms to the low end, and 1200 ms at the high end. The PSI marginal method employed a Weibull function as a model for the psychometric function. It considered SSDs ranging from 50 to 1200 ms in steps of 50 ms, and considered those same values as possible threshold parameters. The slope parameters considered were ranged from 1 to 13 in steps of 1. Error rates considered ranged from 0 to 0.5 in steps of 0.05.

The order of the two blocks was counterbalanced, each run took approximately 30 minutes, containing in total 300 trials: for each of the four foreknowledge-side conditions, there were 17 Change-trials and 51 Go-trials (25% Change-trials per condition), making 272 trials. Additionally, there were 28 certain-Go trials. In both runs, after approximately 75 trials, participants could take a break. The order of trials was quasi-randomized, with the restrictions that no more than 3 Change-trials followed each other, that the first 6 trials of a run were Go-trials, and that the first trial after a break was a Go-trial. The trial list in the second run was the reverse of the trial list in the first run, with the actual list used for first and second run being counterbalanced over participants. Between the two runs, there was a longer break during which demographic- and handedness information was collected.

#### Comparison: Slowing versus non-slowing

In order to compare the performance of slowing and non-slowing participants, all participants were grouped based on the PSI method block. Participants could show both progressive slowing during the course of the block as well as slowed reactions from the outset. To take both kinds of slowing into account during grouping, a plot including all participants’ behavior was created, with the slope of Go-RTs throughout the block on the x-axis and the Go-RTs’ intercept on the y-axis. The two groups were created by choosing a separating diagonal resulting in two roughly similarly sized groups. A visualization of this grouping can be found in S2 Fig.

### Results

**Fig 1:**
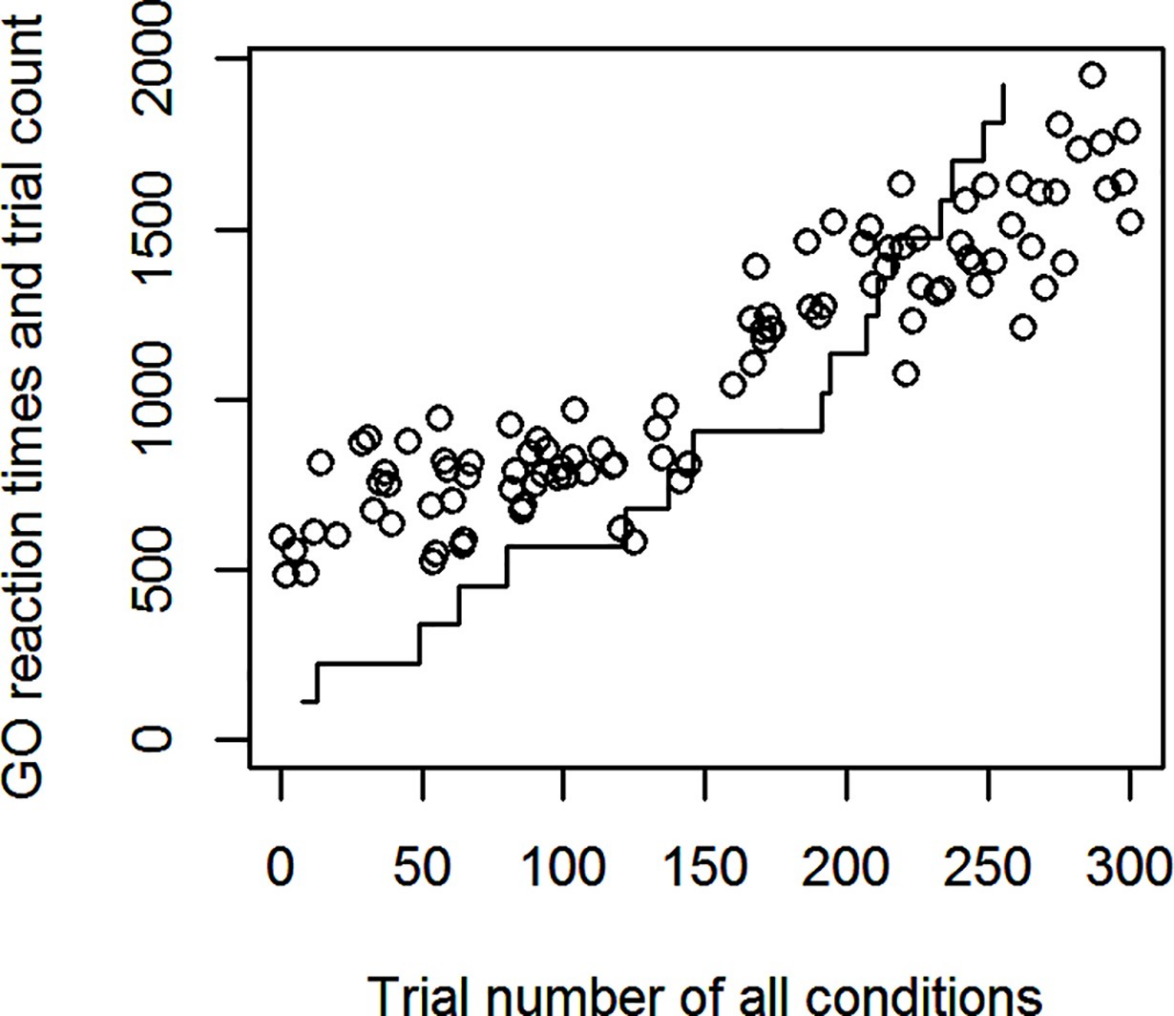
Go-RT slowing in Experiment 1. A participant’s Go-RTs for one condition. The line indicates the increasing number of Change-trials in that condition, the circles are Go-RTs.

**Fig 2:**
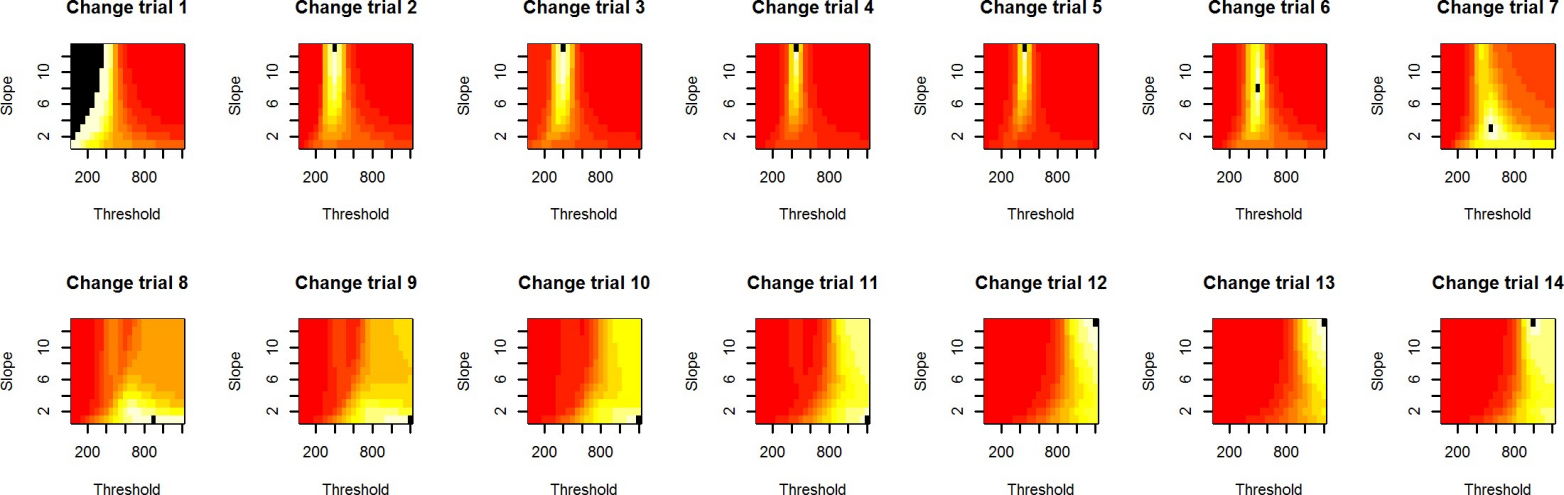
Likelihood estimates during Experiment 1. The PSI marginal method’s posterior distributions for the example participant. Probabilities are averaged over the error rate dimension, colors indicate the probability of a parameter combination, the point of maximum probability is indicated in black. Colors range from minimum to maximum probability per distribution. Around Change-trial 9, the most likely threshold parameter reaches the boundaries of the method’s parameter space.

Experiment 1 was mostly explorative in nature to elucidate the behavior of the PSI marginal method in a task involving Go-response slowing. See Fig 1 for an example participant with gradual Go-response slowing in the PSI marginal method’s block. Since the maximum SSD offered was 1200 ms, the Go-RTs of this participant exceeded this SSD after about half the experimental run. In terms of the two methods’ response to Go-RT slowing, the behavior illustrated in Fig 1 causes a problem for the PSI method. As can be seen in Fig 2, when Go-RTs exceed the maximum threshold considered by the algorithm (at Change-trial 9), the critical SSD (equal to the threshold parameter) receiving the highest probability reaches the upper end of the parameter space, in this case 1200 ms, and the method is not able to estimate the participant’s true critical SSD. In effect, the PSI marginal method is not able to keep up with the slowed Go-RTs. Notice that the SSRT is ultimately computed with the expected parameter combination, not the maximum probability parameters, but nevertheless reaching the boundary of the parameter space invalidates the method’s SSRT estimates. Furthermore, when the mean Go-RT has slowed down considerably, the critical SSD estimated by the method is, after a sufficient number of trials, based on this slowed Go-RT. However, when computing the SSRT, the mean Go-RT of the entire block is usually used. Thus, the critical SSD subtracted from the mean Go-RT might only be based on a slowed subset of Go-RTs toward the end of the experimental block. Although we only tested the PSI marginal method, both of these problems equally exist for the original PSI method.

**Fig 3:**
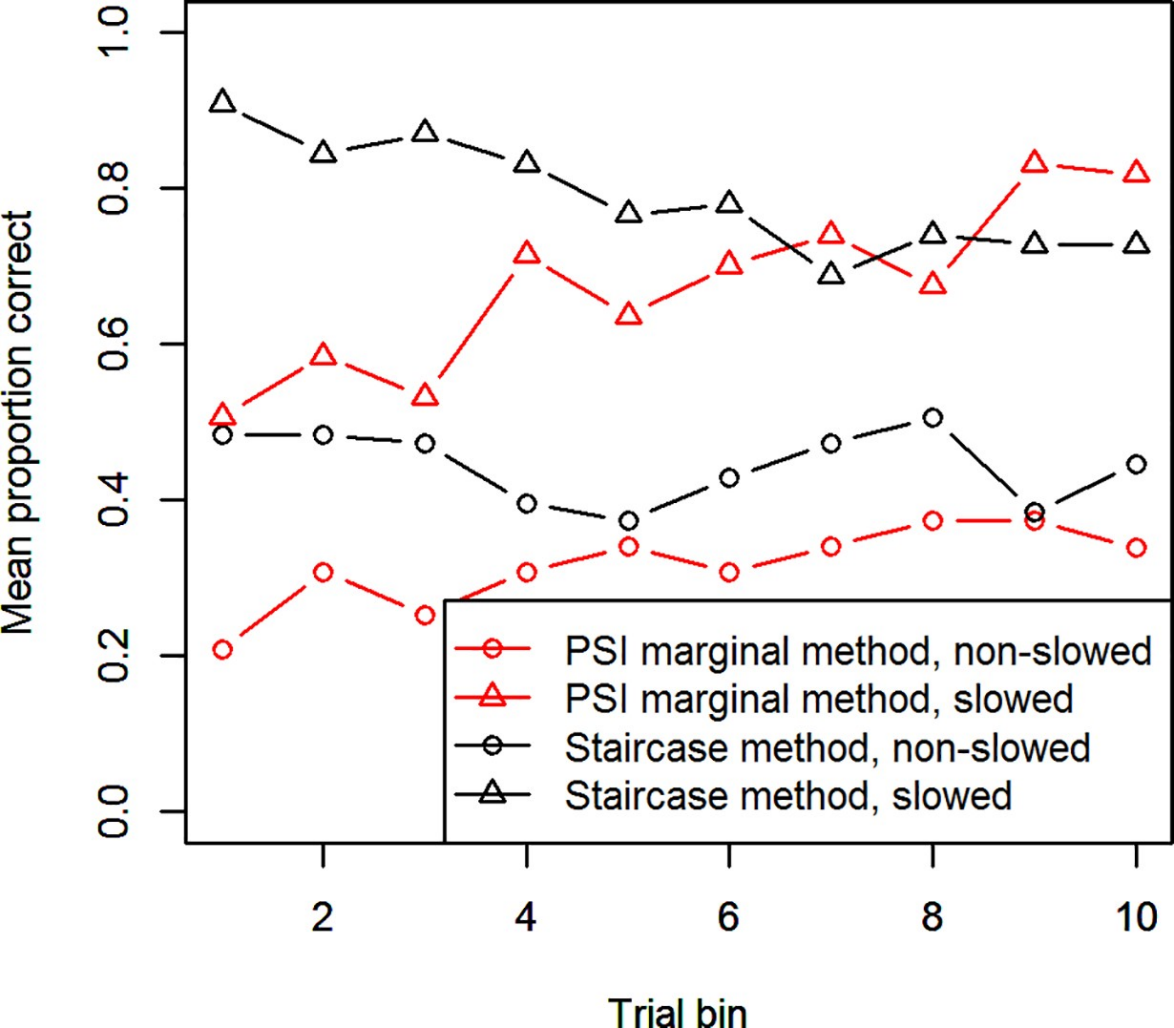
Accuracy throughout Experiment 1. The proportion of successful Change-trials (averaged over bins of 8 trials) of the PSI marginal and Staircase methods. Participants were split into two groups based on whether they showed substantial Go-response slowing.

A further consequence arising from response slowing in the Stop-signal task is the overall accuracy of the participants. Although the staircase method is designed to converge on an accuracy of around 0.5 and can to some degree “follow” the participant’s slowed Go-responses, if responses are being slowed down too quickly, they can escape the staircase and allow the participant to attain an accuracy substantially higher than 0.5. This is especially true if the experiment contains multiple conditions and thus, the amount of response slowing between the different conditions’ interleaved trials is larger. However, an accuracy of around 0.5 is necessary for an accurate computation of the SSRT. Although the PSI marginal method is not explicitly designed to converge at 0.5, it also usually settles around this value. In Fig 3, the proportion of successful change trials is plotted, averaged over bins of 8 Change-trials, for the two different methods and with participants split into two groups, based on whether they showed substantial Go-response slowing. From the figure it becomes clear that only the staircase method for non-slowing participants was able to maintain an accuracy of about 0.5 throughout the experiment. Slowing participants were able to achieve a substantially higher accuracy, for both the staircase and PSI marginal method. Non-slowing participants for the PSI marginal method, on the other hand, stayed below 0.5 performance for the length of the experiment.

What is striking, furthermore, is that in the PSI marginal method’s block, slowed subjects initially achieve a higher accuracy (around 0.5) than non-slowing subjects (0.2). This is owed to the extensive parameter range for critical SSDs (50 ms– 1200 ms) we used in order to allow some response slowing. Before gaining knowledge about a participant’s behavior, the method tends to choose SSDs from the center of its parameter range, which in this case is between 600 and 650 ms, since these are most informative. These SSDs are already too large for non-slowing subjects, leading to their poor performance, while slowing subjects are better able to deal with these trials.

We have included further visualization of the relation between SSRT estimates of the staircase- and PSI method in the supplementary material (S3 Fig).

Since it is clear the PSI marginal method has considerable problems with participants slowing their Go-responses in the Stop-signal task, we propose the PSI adjusted method, which keeps a running estimate of the current mean Go-RT during the experiment. This adjusted method does not estimate the critical SSD, but the difference between estimated mean Go-RT and the critical SSD, thus in effect estimating SSRT directly. In this way, the method’s estimated SSRT is always based on the current mean Go-RT, and also does not change substantially over the course of the experiment, thus not reaching the boundaries of the considered parameters. Additionally, even dramatic Go-response slowing should not be able to cause an accuracy substantially larger than 0.5.

## The PSI adjusted method

In the following the PSI adjusted method, which can also deal with slowed Go-RTs, will be explained in more detail. A participant’s performance is now characterized by a three-dimensional vector *ω* = (*SSRT*_*participant*_,*Slope*_*participant*_,*ErrorRate*_*participant*_). Thus, we estimate SSRT directly, instead of the threshold parameter. The relation between SSRT and threshold is:

Threshold=meanGoRT−SSRT (11)

Since the psychometric function is defined in terms of the threshold parameter, we use Eq 11 to convert a given SSRT value to its corresponding threshold value. In order to do that, we need an estimate of the participant’s mean Go-RT. Since during response slowing, the Go-RT changes throughout the task, the first step in each Stop-trial is to estimate the current mean Go-RT. This is done via linear regression: over a sliding window including the last several Go-trials, the Go-RTs are regressed on their respective trial numbers. The resulting linear regression equation is used to predict the Go-RT of the current trial, which serves as an estimate of meanGo-RT in Eq 11.

The logic behind this is that a participant’s SSRT is a characteristic not changing with response slowing. The slowing only affects the threshold parameter, which indicates the SSD at which *p(error)* = 0.5. This threshold trails behind the continuously slowing Go-RT, while the distance between the Go-RT and the threshold (the SSRT) remains constant. By estimating the changing Go-RT and adjusting the threshold parameter for it, we can estimate the unchanging SSRT.

After having estimated the current Go-RT at the outset of the Stop-trial, we also need to adjust our SSD range. Until now we have not touched upon the range of SSDs to choose from, but since we now adjust for slowing Go-RTs, we have to ensure that the SSDs that can be offered in our Stop-trial match the range of threshold values that are possible under our current estimate of the Go-RT and the range of SSRT parameters in our parameter space. On each Stop-trial, we let SSDs range from

meanGoRT−max(SSRT)tomeanGoRT−min(SSRT)

where min(*SSRT*) and max(*SSRT*) are the minimum and maximum SSRTs of our parameter space. In practice, we also truncate negative SSDs to 0. After these adjustments at the start of a Stop-trial, the method follows the PSI marginal method outlined earlier, except that all distributions are now defined over *ω* parameter space.

In order to compare the properties of our PSI adjusted method during the Stop-signal task to the PSI marginal method as well as the classic staircase method, we ran three sets of simulations. The first set is very similar to those presented by Livesey & Livesey [4] and investigates the qualities of the SSRTs estimated by the different methods under the condition of stationary Go-RTs. The second and third set of simulations investigate the effects of Go-RT slowing on the different methods.

## Simulations 1: Constant reaction times

### Methods

#### Simulations

In order to quantify and compare the performance of our PSI adjusted method, the PSI marginal method and the staircase method, we simulated several Stop-signal experiments. In each, per trial human performance was simulated by randomly drawing a Go-RT from an ex-Gaussian distribution with parameters μ = 360, σ = 40 and ν = 40. These parameters remained constant over the experiment. If it was a Go-trial, this was the Go-RT. If it was a Stop-trial, the outcome of the trial was taken to be an erroneous response if the Go-RT was smaller than the simulated SSD plus the participant’s SSRT; otherwise it was a successfully inhibited trial. In each experiment there was a fixed error rate, which governed how many percent of trials a participant’s response was inverted, i.e. successful inhibition becoming erroneous response and vice versa. The error rate varied across experiments but was the same for all participants. Each experiment contained 100 Stop-trials and 200 Go-trials. In each experiment, 41 participants were simulated, each with a different SSRT ranging from 50 to 250 in steps of 5 ms. In each experiment, three different methods ran in parallel (classic staircase method, the PSI marginal method, our PSI adjusted method), each determining on its own which SSD should be presented next, and each receiving responses based on separately drawn Go-RTs. After each Stop-trial, two Go-trials were simulated, and all participants shared the same Go-RT per Go-trial. Three different error rates of 0, 0.05, and 0.1 were simulated, with 50 experiments per error rate. The performance measures for the four different methods were averaged over error rates and experiments. The staircase started at an SSD of 250 ms, was increased or reduced by 50 ms after successful or unsuccessful Stop-trials, respectively, and was limited to 0 ms at the low end. For the staircase, the SSRT was computed in two ways, once with the mean method and once with the integration method. All simulations were programmed using the R programming language and environment (R Core Team, 2017).

In contrast to the behavioral experiment, in the simulations we used a function of the form

$$p(\mathrm{error})=\mathrm{ErrorRate}+\frac{1-2*\mathrm{ErrorRate}}{1+e^{-\mathrm{Slope}*(\mathrm{SSD}-\mathrm{Threshold})}}$$ (12)

to model the psychophysical performance. This function follows the form of the logistic function but lets the error rate govern both the minimum and maximum the function can take on. The PSI marginal method used possible SSDs ranging from 0 to 500 ms in steps of 50 ms. The threshold parameters estimated as the critical SSDs ranged from 0 to 500 ms in steps of 5 ms, the slope parameters were 0.003, 0.0052, 0.01, 0.019, 0.029 and 0.04, the error rates ranged from 0 to 0.3 by steps of 0.05. Our PSI adjusted method estimated SSRTs ranging from -100 to 400 ms in steps of 5 ms (thus being centered on the actually simulated SSRTs, exceeding them by 150 ms to both sides, similar to the PSI marginal method under the stated Go-RT distribution parameters). The slope values and error rates were identical to the PSI marginal method. SSDs were updated each trial based on the current predicted Go-RT estimate, as described earlier, ranging from the current predicted Go-RT minus the longest SSRT estimate, until the predicted Go-RT minus the shortest SSRT estimate, in steps of 50 ms rounded to the nearest full 50 ms and set to 0 ms for negative values. The running estimate of the Go-RT was computed by linearly regressing the Go-RTs of the last several Go-trials (at least 15, at most 40; covering the last 7–20 Stop-trials) on their trial’s number, and then predicting the upcoming Go-RT from the linear regression equation. If not enough trials had been passed to make a linear prediction, a scalar estimate of the Go-RT was used, set at 400 ms.

#### Analysis

Per experiment, we computed how the SSRT estimate of each method developed over the course of the experiment, so per trial we computed each method’s estimate of the SSRT up to that trial, using the Go-RTs obtained up to that trial. For the staircase method, SSRTs were computed both according to the mean- and the integration method. We then computed three measures of performance for these SSRT estimates, similar to Livesey & Livesey [4]: the correlation of a method’s SSRT estimate with the actually simulated SSRT (over participants), the mean absolute deviation of SSRT estimate from the simulated SSRT (averaged over participants), and the slope of the linear regression line relating the different participants’ SSRTs to the estimated SSRTs. These parameters, developing over the course of each experiment, were then averaged over the different simulated experiments.

### Results

**Fig 4:**
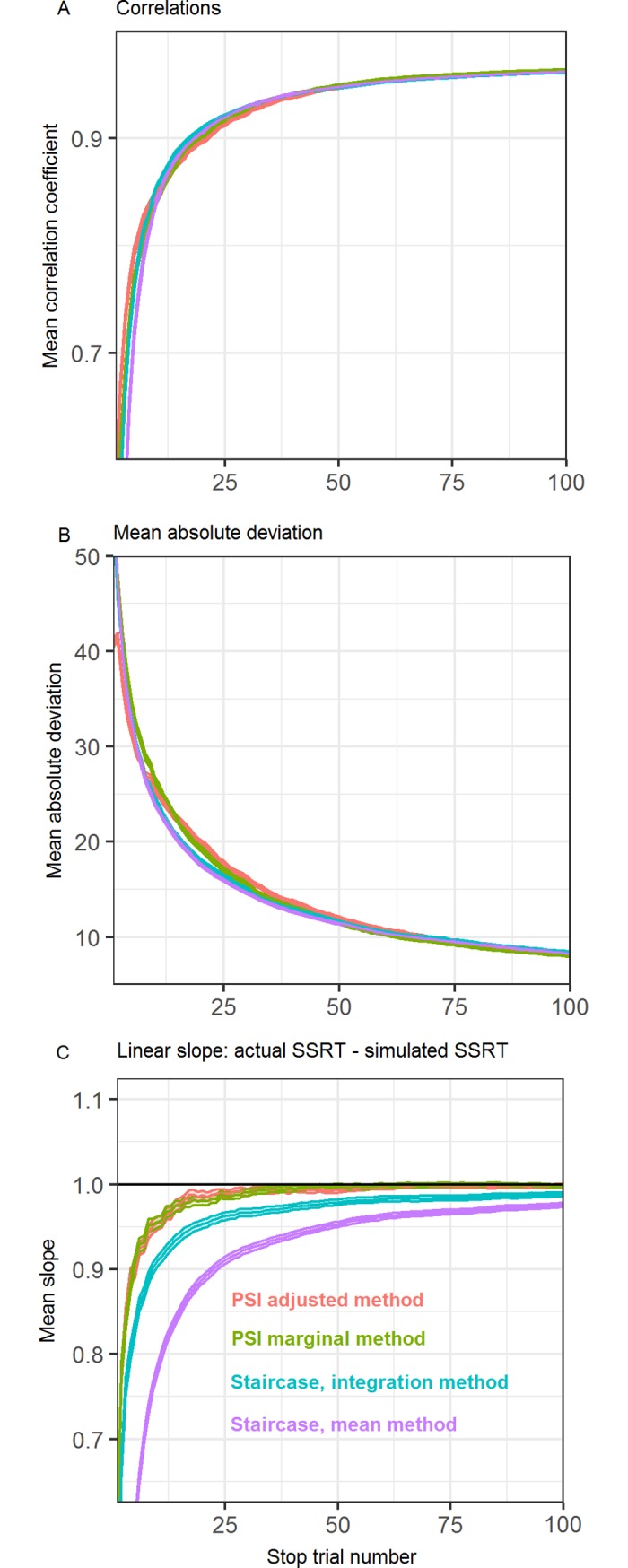
Different methods’ performance with stationary GO-RTs. Performance of the PSI marginal method, PSI adjusted method, and the staircase method with stationary Go-RTs. (A) Correlations of estimated SSRTs with simulated SSRTs. (B) Mean absolute deviation between estimated and simulated SSRTs. (C) Linear slope between estimated and simulated SSRTs. Filled areas indicate the standard error of the mean over the 150 simulated experiments.

As Fig 4A shows, the correlations between all four methods’ SSRT estimates and the true SSRTs develop similarly over the course of the experiment, reaching the 0.9 mark around 18 trials and further increasing from there. No substantial differences between the methods are obvious.

Fig 4B shows the mean absolute deviation of SSRT estimates for the different methods. Again, the time course over the experiment is generally similar for the different methods, with only a small underperformance of the PSI methods between trials ten to 25, corresponding to a mean absolute deviation of around 2.5 ms after 12 trials.

The most interesting result is depicted in Fig 4C, where the linear slope between a method’s SSRT estimate and the true SSRT is plotted over the course of the experiment. This can be seen as an index of biased SSRT estimation, with slopes smaller than one meaning lower SSRTs are being overestimated or higher SSRTs being underestimated, or both. An unbiased estimation would result in slopes of one. As can be seen, there is a clear ordering of performance for the different methods, with the PSI methods performing best and equally well, the staircase integration method performing worse and the staircase mean method performing worst. The differences in performance are larger in the lower trial numbers. Since the staircase- and PSI methods settle on different ultimate slope values, it will take progressively longer for the staircase methods to reach the same slope values as the PSI methods the closer that value is to one. For example, it takes the staircase integration method 4 trials longer than the PSI methods to reach a slope of 0.9, but 11 trials longer to reach a slope of 0.95, and 21 trials longer to reach a slope of 0.97.

## Simulations 2: Increasing reaction times

### Methods

In most respects, the second set of simulations is identical the those presented thus far, except that now only one error rate was simulated (0.05), and that Go-RTs now slowed down over the course of the experiment. Three different extents of slowing were simulated: Go-RTs slowing 5 ms per Stop-trial, 10 ms or 15 ms (the first two extents corresponding roughly to the degrees of slowing simulated by Verbruggen, Chambers and Logan [3] and the third representing a more severe kind of slowing that can be found in experiments containing multiple conditions). Slowing was simulated by increasing the μ parameter of the ex-Gaussian distribution used to draw Go-RTs from. The results will be presented averaged over the different degrees of slowing, the results split up for the different amounts of slowing can be found in the supplementary materials (S4 Fig).

### Results

**Fig 5:**
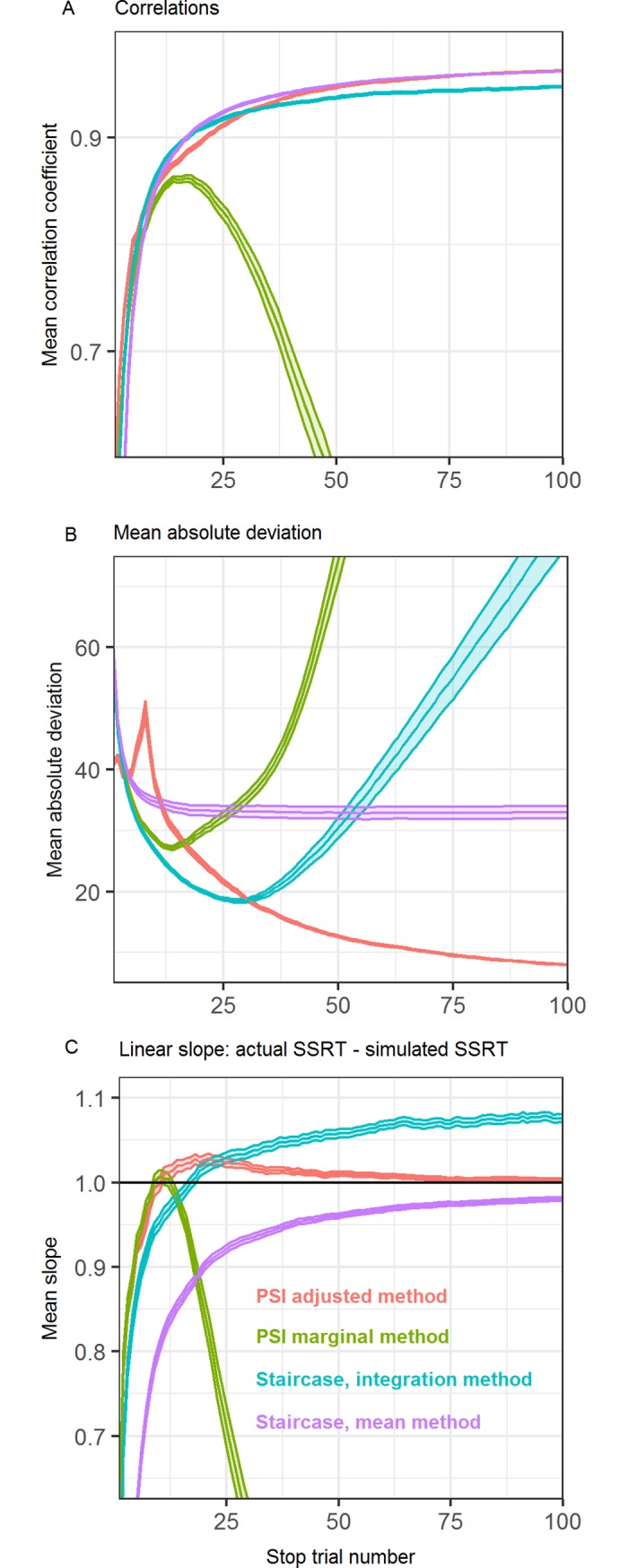
Different methods’ performance with slowing GO-RTs. Performance of the PSI marginal method, PSI adjusted method, and the staircase method with increasing Go-RTs. (A) Correlations (B) Mean absolute deviation (C) Linear slope. Filled areas indicate the standard error of the mean.

As Fig 5A shows, when Go-responses are slowed over the course of the experiment, the correlations for the staircase and PSI adjusted method are fairly similar to when responses are not being slowed. The PSI marginal method’s performance, however, deteriorates from around trial 12 onwards, due to it not being able to react to the slowing Go-responses (and changing critical SSDs). Not visible in the graphic, the correlation coefficients for the PSI marginal method decrease until 0.2 at trial 100.

In Fig 5B, it can be seen that the staircase-integration method, the staircase-mean method and the PSI marginal method all at some point deteriorate in performance due to not being able to keep up with the slowing Go-RTs. The staircase-integration method performs best until around trial 30, while the staircase-mean method stops improving at a mean absolute deviation of around 35 ms. While initially performing worse than the staircase-integration method, the PSI adjusted method is able to keep up with slowing Go-RTs and steadily decrease absolute deviation. The deviations for the staircase- and PSI marginal method eventually increase to around 80 and 275 ms, respectively.

In terms of the slope between estimated and actual SSRTs, the staircase-mean method stays consistently below one. The PSI marginal method deteriorates in performance around trial 12. Not shown in the graphic, the slope eventually decreases down to almost 0 at trial 100. The staircase integration method quickly surpasses a slope of one, again not able to keep up with response slowing. The PSI adjusted method quickly rises towards a slope of one and stays there for the remainder of the trials. As can be seen in the supplementary materials (S4 Fig), the stronger the response slowing, the stronger and earlier the adverse effects on SSRT estimation occur.

Generally, while in the case of constant RTs (simulations 1) the PSI methods simply attain a given degree of precision in SSRT estimates in fewer trials than the staircase methods, with increasing Go-RT slowing it becomes more a question of being able to deal with the slowing at all.

## Simulations 3: “Pure” slowing

### Methods

In simulations 2, we saw that Go-response slowing has a clear detrimental effect on the PSI marginal method, which the PSI adjusted method can correct for. However, this leaves open the question of whether this decrement in performance results simply from the RTs increasing (“pure” slowing), or whether the cause of the problem is the behavior of the participant reaching and crossing the boundaries of the method’s parameter space, specifically the threshold parameter’s boundaries. These two issues are separable, since pure slowing can occur while the participant’s behavior is still well within the parameter space, and since a certain degree of pure slowing can be accommodated by simply increasing the range of threshold parameters available to the PSI marginal method.

To investigate the effect of pure slowing on the PSI marginal and PSI adjusted methods in the absence of reaching the parameter space’s boundaries, we performed an additional set of simulations building on simulations 2.

The simulations were generally identical to simulations 2, again with 3 different degrees of response slowing being simulated (5, 10 and 15 ms per Stop-trial), but now for each participant only the PSI marginal method and PSI adjusted method ran in parallel. Both methods worked as described in simulation 2, except that now, the parameter space of the PSI marginal method was changed in a way that until Stop-trial 100, all participants’ behavior stayed within the parameter space despite the Go-RTs increasing. For example, with a Go-response slowing of 5 ms per Stop-trial, after 100 Stop-trials, the mean Go-RT lies at the initial 400 ms + 500 ms = 900 ms. Since the critical SSD = Go-RT–SSRT, and since the fastest SSRT simulated was 50 ms, at the end of the 100 trials the largest critical SSD of the simulated participants lies at 850 ms. To accommodate for this increase of the critical SSD (and thus, the increasing threshold parameter of the PSI marginal method), we set the threshold parameter space of the PSI marginal method to range from 0 to 1000 ms (in steps of 5 ms). Since the smallest critical SSD at the start of the task lies at 400 ms– 250 ms = 150 ms, the 0 ms lower end of the parameter space leaves a buffer of 150 ms. Similarly, the 1000 ms upper end exceeds the largest critical SSD at the end of the experiment by 150 ms. This principle was adhered to by all three simulated degrees of response slowing. Thus, for a slowing of 10 ms per Stop-trial, the threshold parameter space for the PSI marginal method ranged from 0 to 1500 ms in steps of 5 ms, while for a slowing of 15 ms per Stop-trial, the PSI marginal method’s parameter space ranged from 0 to 2000 ms in steps of 5 ms. The possible SSDs always spanned the same range in steps of 50 ms. The PSI adjusted method did not need an adjusted parameter space.

To also see the effect of crossing the parameter space’s boundaries, the tasks were simulated further than the initial 100 Stop-trials, until the point when all participants had left the PSI marginal method’s parameter space. For 5 ms of slowing per Stop-trial, this resulted in 170 simulated trials. For 10 and 15 ms of slowing, 135 and 124 trials were simulated respectively.

### Results

**Fig 6:**
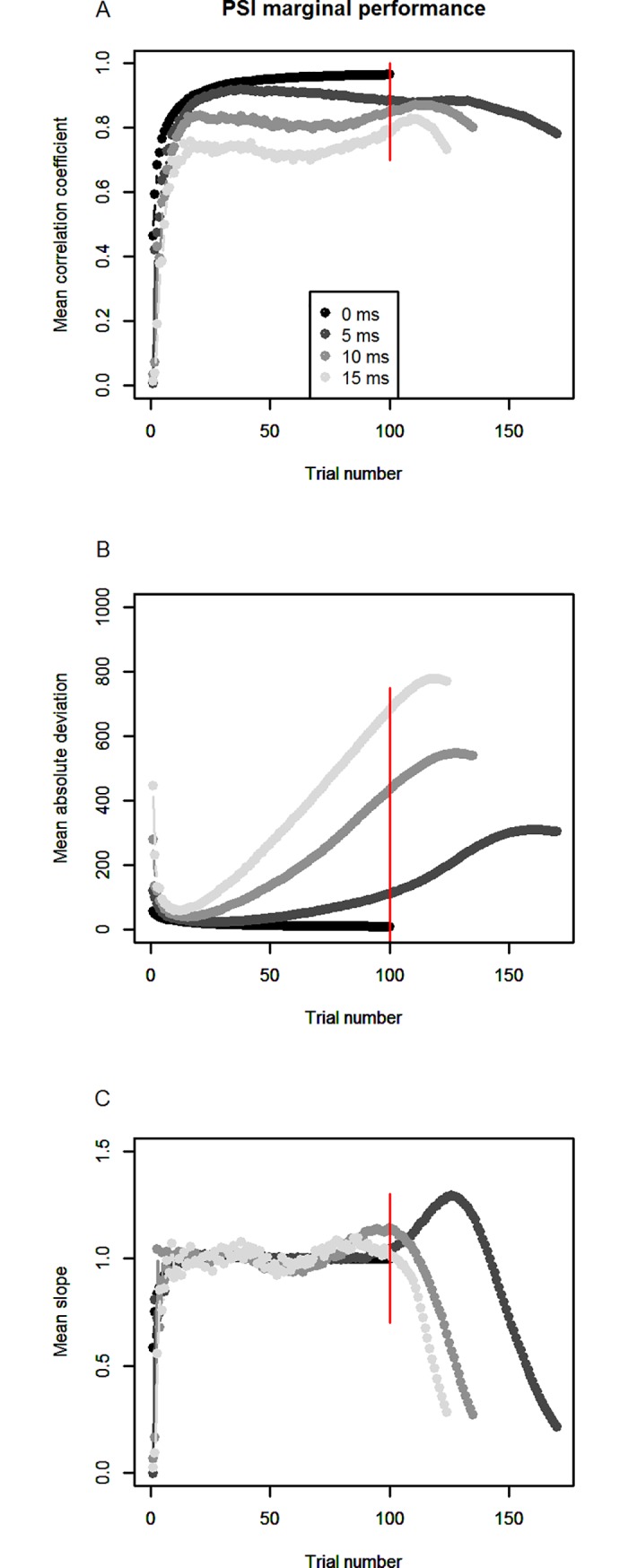
PSI marginal method’s performance for different degrees of slowing inside parameter space. Panel A shows correlation coefficients between simulated and estimated SSRTs, panel B shows mean absolute deviation between simulated and estimated SSRTs, panel C shows mean linear slope relating simulated and estimated SSRTs. Colors indicate different degrees of response slowing. Until trial 100 (red line), all simulated participants are inside the method’s respective parameter space.

**Fig 7:**
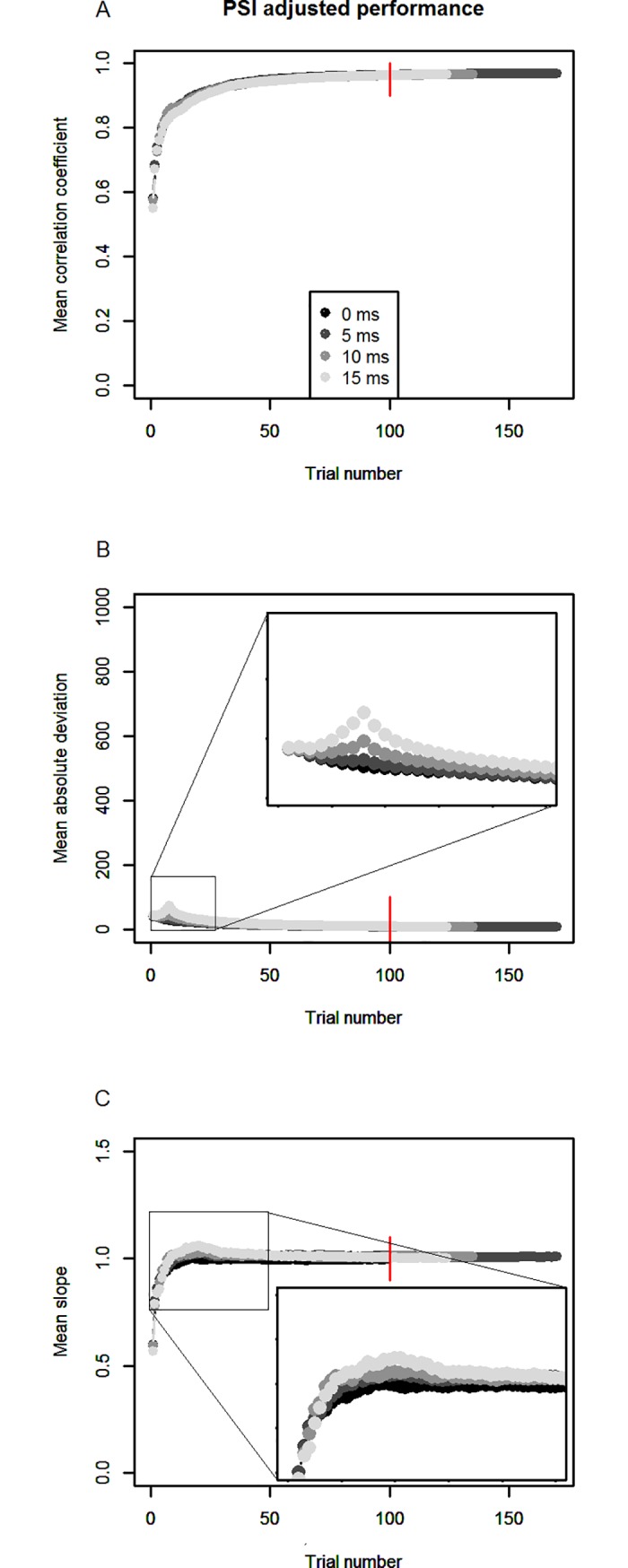
PSI adjusted method’s performance for different degrees of slowing inside parameter space. As in Fig 6, but now the performance of the PSI adjusted method is depicted.

**Fig 8:**
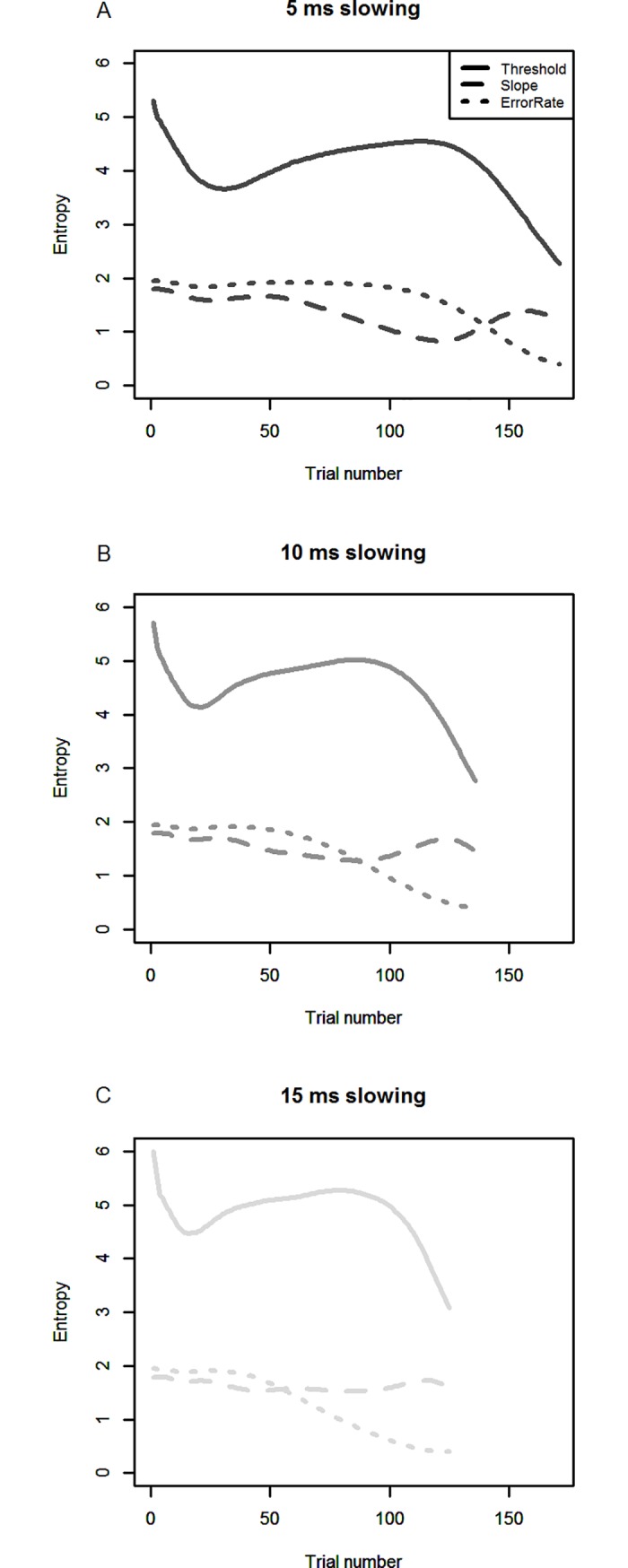
Marginal entropy of the PSI marginal method over the course of the task. The marginal entropies of the threshold, slope and error rate parameters are shown, over the course of the task, for the PSI marginal method. Line pattern indicates which parameter’s entropy is shown. The upper panel shows the results for 5 ms of slowing per Stop-trial, the middle panel for 10 ms, and the lower panel for 15 ms.

The results can be seen in Figs 6–8. Correlations, mean absolute deviations and linear slopes were computed as for simulations 1 and 2. To compare the performance of the two methods to the case of no Go-response slowing, we also show the results of simulation 1 again (in black), in which Go-RTs were constant.

As can be seen in Fig 6, the effects of Go-RT slowing on the PSI marginal method’s performance are already apparent before the boundaries of the parameter space are reached (trial 1 to 100): the stronger the response slowing, the lower the correlation between simulated and estimated SSRTs (Fig 6A), ranging from around 0.9 between trials 25 and 75 for 5 ms slowing, to around 0.75 for 15 ms of slowing. When the boundaries of the parameter space are reached (and crossed; after the red line), correlation coefficients start to decrease more strongly.

The same effect of response slowing is apparent for mean absolute deviation (B) between simulated and estimated SSRTs: the stronger Go-RT slowing, the larger the deviation becomes, even before reaching the parameter space’s boundaries. At trial 100, when the end of parameter space is reached, the mean absolute deviation for 5 ms of slowing lies at around 100 ms; for 10 ms, it lies at around 430 ms; for 15 ms, it lies just below 700 ms. Not shown here, the results also indicate that the increased absolute deviation during the first 100 trials is mainly due to incorrect estimation of the Go-RT and only to a lesser extent the result of erroneous estimation of the critical SSD.

In the linear slopes between simulated and estimated SSRTs (Fig 6C), the pattern is not so clear while participants remain inside parameter space: while slopes seem to slightly oscillate around 1, especially for stronger response slowing, the marked deterioration of performance for the PSI marginal method only begins after the threshold parameter space was left, with slopes sharply declining.

As visible in Fig 7, the PSI adjusted method is able deal with response slowing both when participants remain inside the parameter space as well as when they leave parameter space. In mean absolute deviation as well as linear slopes, some gradation is visible, with stronger response slowing leading the decreased performance (insets), but the size of these effects is not comparable to the PSI marginal method’s deterioration of performance.

In Fig 8, we show the entropy of the PSI marginal method’s posterior distributions over the course of the simulated experiments, separately for 5, 10 and 15 ms response slowing per Stop-trial. Furthermore, for each degree of response slowing, we separately show the entropy for threshold parameter (summed over slope and error rate), slope (summed over threshold and error rate) as well as error rate (summed over threshold and slope parameters). The parameter dominating the overall entropy, which can be envisioned as the sum of all three entropies, is the threshold parameter. It is clear that after a short drop in entropy for the threshold parameter (until trial 25 for 5 ms), the value increases gradually until trial 100. Starting at trial 100, when the boundaries of the parameter space are hit, overall entropy decreases again, which is largely driven by the threshold parameter. This shows that while response slowing leads to an increase in entropy when the parameter space has not been left yet, especially for the threshold parameter, the event of hitting the boundaries of parameter space and leaving it leads to decreased entropy, most likely due to the probabilities of the posterior distributions concentrating at the outer edges of parameter space.

## Behavioral experiment 2: The PSI adjusted method

As the results of Experiment 1 showed, the presence of strong response slowing caused different problems for the PSI marginal method. After finding a good performance of the PSI adjusted method in the simulations, we wanted to put the method to the test in human participants and see whether these problems persisted. As in Experiment 1, a group of participants completed two blocks of the Stop-change task, one with SSDs controlled by the staircase method, and one controlled by the PSI adjusted method described above.

### Methods

#### Participants

Participants were 20 students or former students (M_age_ = 23.4 ± 3.2, 6 male, 3 left-handed) from the same population as Experiment 1, with normal or corrected-to-normal vision and no history of neurological or mental illness. Participants were paid 15 € for their participation. Informed consent procedures were the same as in Experiment 1, the local ethics committee of the university hospital of the RWTH Aachen had approved the experiment.

#### Task

Participants completed the same Stop-change task described in Experiment 1.

#### Procedure

Again, participants started with a training run, followed by two consecutive runs of the Stop-change task, one controlled by the staircase procedure and one controlled by the PSI adjusted method. The settings for the staircase procedure were generally identical to Experiment 1, with the lowest SSD now 0 ms and the largest SSD now 2300. The largest SSD was increased since the PSI adjusted method now also allowed for further response slowing compared to Experiment 1. The PSI adjusted method used the same psychometric function as described for the simulations. The SSRTs considered as parameters ranged from 0 to 600 ms in 24 equally sized steps, rounded to the nearest millisecond. The slope parameters considered were 0.003, 0.0052, 0.01, 0.019, 0.029 and 0.04, and the error rates considered ranged from 0 to 0.5 in steps of 0.05. The possible SSDs were updated each trial as described above, and the current predicted Go-RT was estimated based on the last 15 to 40 Go-trials. Less than 40 trials were usually used at the beginning of the task, when not enough Go-RTs had been collected yet. The certain-Go condition was excluded from Go-RT prediction, since Go-RTs in this condition are known to differ from those in the change-conditions. Although the possible SSDs computed in this fashion can increase indefinitely, they were capped at 2300 ms. At the beginning of the PSI method’s run, the Go-RTs from the preceding block, either training or staircase, were used to initially predict Go-RT.

All other procedures were kept identical to Experiment 1.

#### SSRT computation

For the staircase block, SSRTs were computed using the integration method, with all Change-trials containing erroneous responses considered signal-respond trials. For the PSI adjusted method, SSRTs were estimated directly, and thus the expected SSRT was derived from the ultimate posterior distribution.

### Results

**Fig 9:**
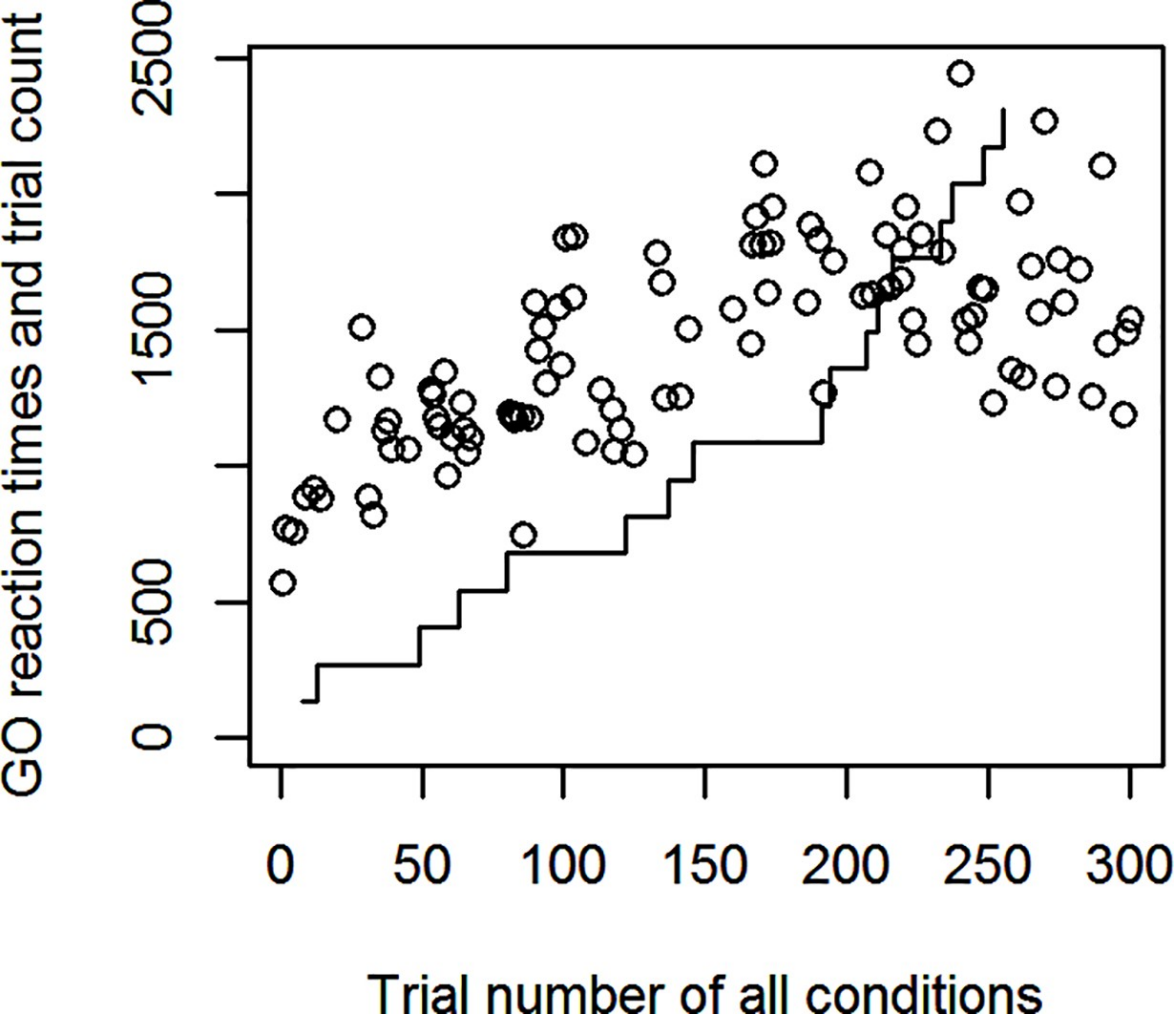
Go-RT slowing in Experiment 2. As Fig 1, a participant’s Go-RTs for one condition in the PSI adjusted method’s block. The line indicates the increasing number of Change-trials, the circles are Go-RTs.

**Fig 10:**
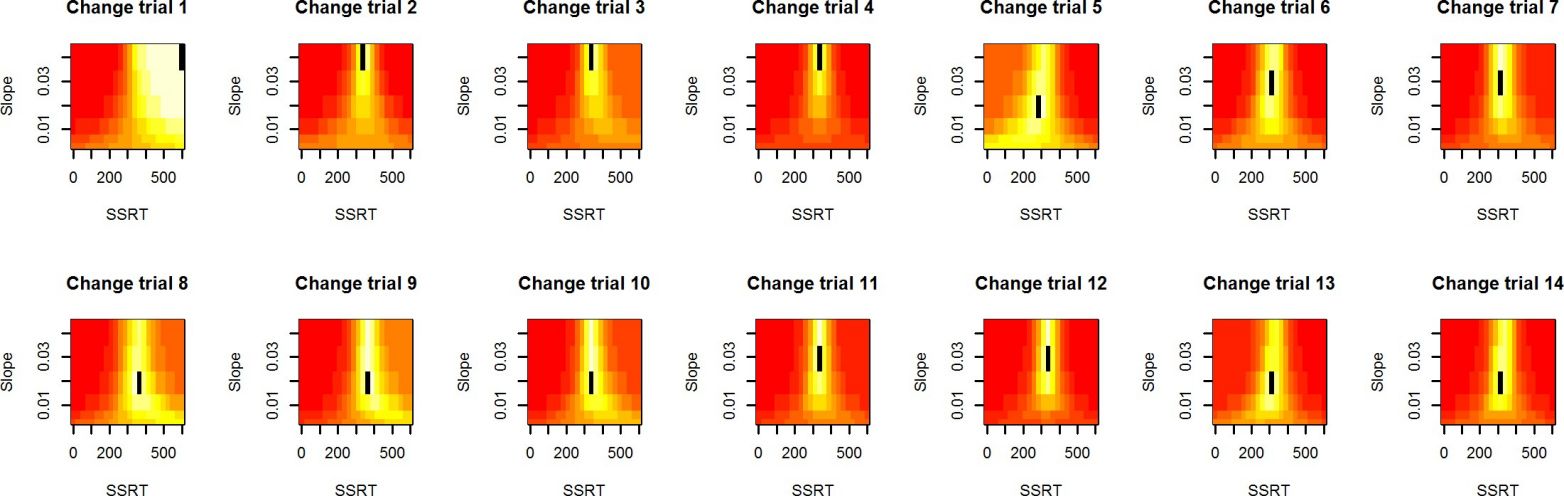
Likelihood estimates during Experiment 2. As Fig 2, the PSI adjusted method’s posterior distribution for the example participant. Colors indicate probability of the parameter combinations, the error rate dimension was averaged over. Despite considerable Go-response slowing, the parameters receiving the highest probability do not reach the upper boundaries of the parameter space.

We present the results of Experiment 2 in a similar manner to Experiment 1. Figs 9 and 10 are taken from a participant showing Go-response slowing similar to the example participant from Experiment 1 (Figs 1 and 2). Even though there is considerable slowing, the SSRT estimate of the PSI adjusted method is fairly stable across change trials, allowing the method to optimally sample SSDs despite Go-responses being slowed.

**Fig 11:**
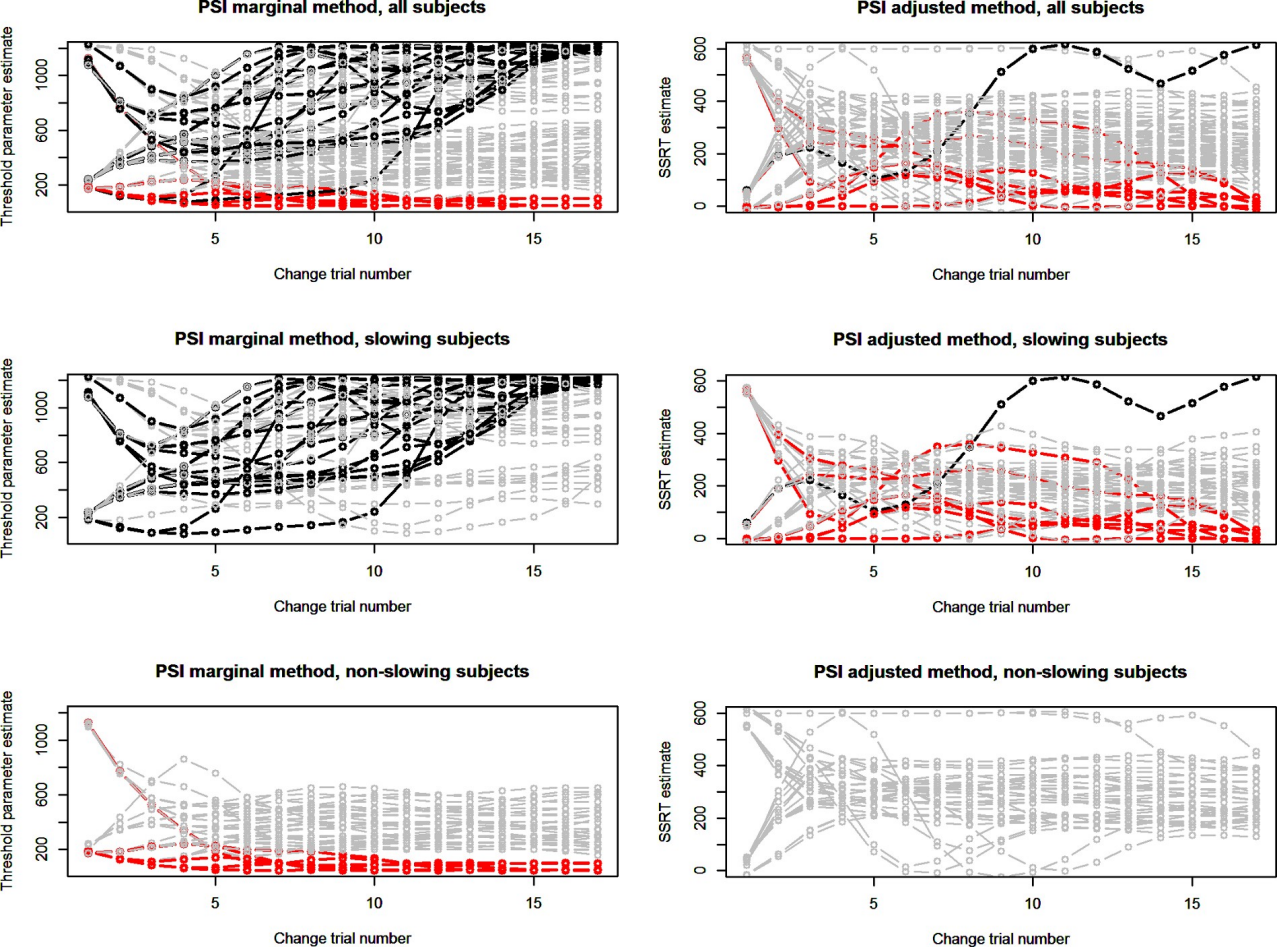
Parameter estimates for Experiment 1 and 2. Highest probability parameters for the PSI marginal method (Experiment 1) and PSI adjusted method (Experiment 2) over the course of the experiment. The participants were split intro groups depending on whether the showed go-response slowing. Every line represents one of the stop-change task’s conditions of one participant; thus, there are four lines per participant. Black lines indicate a parameter reaching the upper end of the parameter space, red lines indicate parameters reaching the lower end. Parameter estimates were slightly smoothed using a cubic spline to facilitate visual inspection. Note that while maximum probability parameters are shown, SSRTs are eventually computed based on the expected parameters.

Fig 11 compares the PSI marginal and PSI adjusted method with respect to their parameter estimates over the course of the experiment. For the PSI marginal method (Experiment 1), there is a considerable number of participants, specifically those slowing their Go-responses, for which the threshold parameter receiving the highest probability in the posterior distribution reaches the method’s upper boundary, thus limiting the method’s ability to estimate the true SSRT. Furthermore, a smaller number of participants reach the lower boundary of the PSI marginal method’s parameter space. These are participants showing particularly fast Go-responses.

While for the PSI marginal method strong response slowing causes a parameter drift toward the upper boundary, in the PSI adjusted method the maximum probability parameters are visibly more stable over time, both for slowing and non-slowing participants. There is, however, a small number of participants in the slowing group whose parameter estimates in the PSI adjusted method drift toward the lower end of the parameter space. There thus appears to be some residual influence of response slowing on parameter estimation. Importantly, this problem is far less pronounced than in the PSI marginal method used in Experiment 1. We come back to the reasons behind this effect in the discussion.

**Fig 12:**
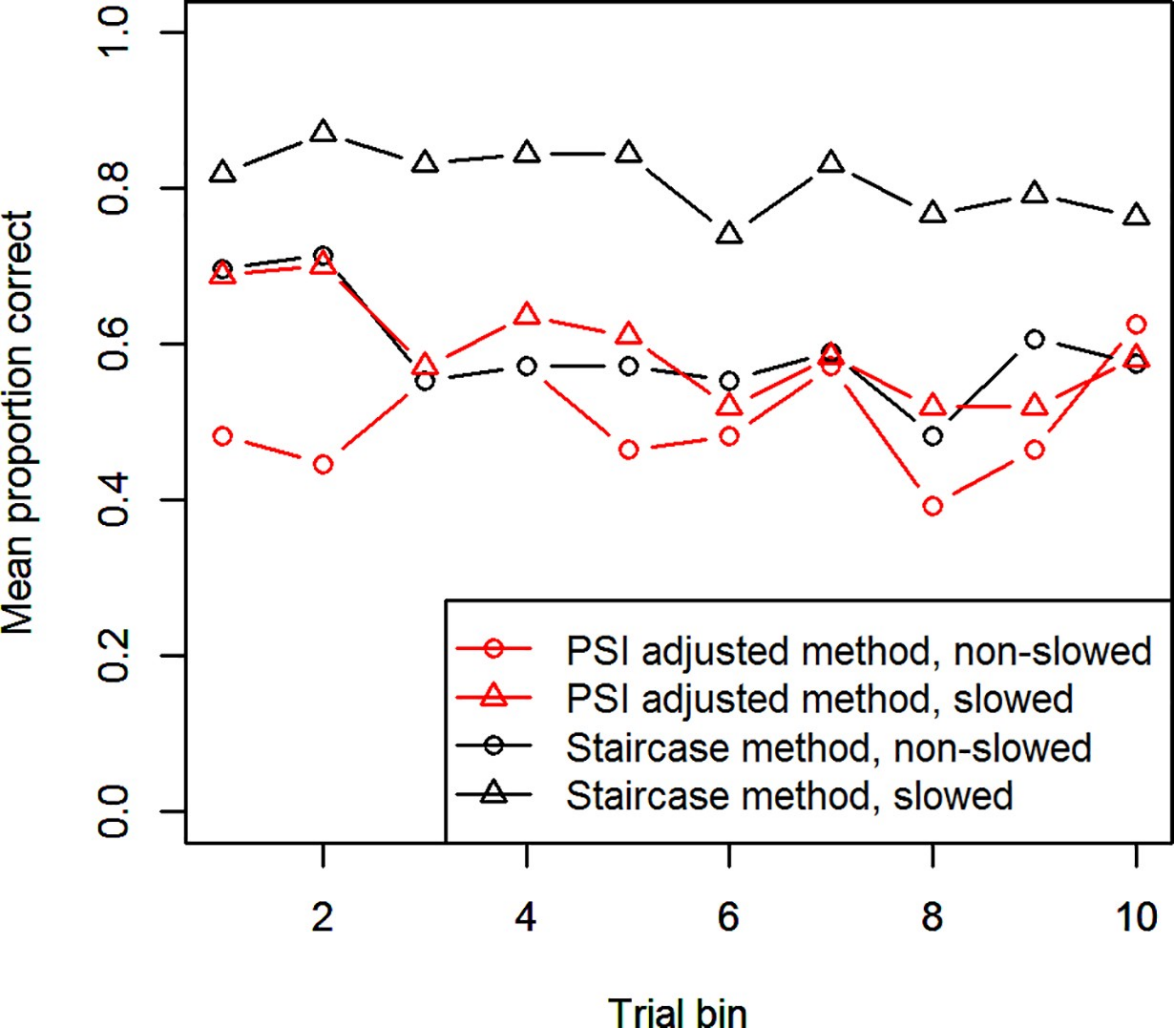
Accuracy throughout Experiment 2. As Fig 3, the proportion of successful Change-trials for the PSI adjusted and Staircase methods, separately for slowed and non-slowed participants.

Another problem we noted for the PSI marginal method and staircase in Experiment 1 was that for slowing participants, accuracy levels substantially deviated from 0.5. As Fig 12 shows, the PSI adjusted method is successful at maintaining accuracy at roughly 0.5 throughout the experiment, for both slowing and non-slowing participants. As in Experiment 1, the staircase method is only able to maintain this intended accuracy level for non-slowing participants.

As for experiment 1, we included further results on the relation between SSRT estimates of the staircase- and PSI method in the supplementary material (S5 Fig).

A further question relevant to the PSI adjusted method is how many trials are optimal when estimating Go-RTs through linear regression. While the up-to 40 trial long window used by us to predict current mean Go-RT appears to have worked fine, we present additional analysis of Go-RTs in supplementary S6 Fig, in which mean and standard deviation of the residuals of the RT prediction are shown, as a function of different window sizes. While the variability of residuals reaches a stable minimum with window sizes larger than 10 to 15 trials for both slowing and non-slowing subjects, the mean residual stays between -1 and 1 ms roughly between window sizes of 1 and 30 trials. Thus, according to our behavioral data, window sizes over 10 to 15 trials, but not substantially larger than 30 trials, are recommended. It should be noted that the optimal window size depends on the timescale and form of changes in RTs, which in turn likely depend on the specific task, such that other numbers of trials might be better suited to predict Go-RT in different tasks.

## Discussion

The original PSI method for the Stop-signal task [4] was proposed to allow optimal sampling of SSDs in Stop-trials, thus allowing shorter experimental lengths. In this article, we showed that the PSI marginal method, and by implication the original PSI method, cannot adequately deal with the kind of response slowing often found in Stop-signal tasks. We proposed a PSI adjusted method that continuously estimates Go-RTs and adjusts for possible slowing, thereby allowing optimal sampling of SSDs despite slowing. In three sets of Stop-signal task simulations, we compared the performance of this adjusted method to the PSI marginal method as well as the classic staircase method. Furthermore, we tested how the PSI adjusted method fares when employed with human participants showing strong Go-response slowing.

From the simulation results, it is clear that when there is no response slowing, the PSI methods offer improved performance in estimating SSRTs, especially for small trial numbers and when unbiased estimation of the spectrum of SSRTs is important. In the face of response slowing, as is common in Stop-signal tasks, it is crucial to correct the PSI method for the changing response speed. When this is achieved, as in our proposed PSI adjusted method, this method can offer improved performance over the staircase method even under considerable response slowing.

Another interesting result most clearly visible in Fig 4 is that the traditional staircase combined with the mean method is considerably worse for estimating SSRTs at low trial numbers than when combined with the integration method. This performance decrement is visible when inspecting the slope between simulated and estimated SSRTs, showing that either smaller SSRTs are overestimated or larger SSRTs are underestimated. The cause of this behavior is that when using the mean method, it is assumed that the SSDs offered up to this point correspond to a performance of 0.5. However, since the staircase takes a certain time to reach that performance level, the SSDs subtracted from the mean Go-RT will introduce a certain bias, which varies depending on the true SSRT of the participant. With the integration method, on the other hand, this bias is corrected for by using not the mean Go-RT, but a percentile of the RT distribution that corresponds to the participant’s behavior.

A further benefit of the PSI methods not discussed so far is that for every point during the experiment, they provide a likelihood distribution over the parameters underlying the participant’s performance, and thus a likelihood distribution over the SSRT estimate at that point. This allows computing a confidence interval of sorts around the updated SSRT estimate after every Stop-trial (similar to the illustration of SSRT estimation in Fig 7). This opens up interesting new experimental opportunities, such as only running a Stop-signal task until a certain level of confidence in SSRT has been reached, or dynamically controlling stimulus presentation based on the SSRTs that are likely or unlikely for a participant.

As shown in the results section of Experiment 2, there appears to be a residual effect of Go-response slowing on SSRT estimation even for the PSI adjusted method. This is likely the result of less than optimal estimation of Go-RTs for this method, particularly an underestimation of Go-RTs. The mechanism behind this is the following: when participants have longer Go-RTs than estimated by the method, they are capable of still stopping for SSDs that would be too long under their actual SSRT, thus leading to a reduced estimated SSRT. Since the PSI adjusted method presented here predicts Go-RTs by linearly regressing previous RTs on trial number, it is clear that any kind of non-linear change in response times cannot be accommodated. Furthermore, the PSI adjusted method only predicts mean Go-RTs, thereby ignoring other properties of the Go-RT distribution, such as variance and skewness, which would be taken into account under the ex-Gaussian parameterization of the RT distribution. As a future improvement to the method, more flexible and behaviorally realistic models of response slowing could be used to better capture changes in RTs throughout the experiment. It stands to reason that this would further improve the statistical properties of the PSI adjusted method.

S3 and S5 Figs show the relationship between the SSRT estimates of the staircase blocks and the PSI method’s blocks, separately for Experiment 1 and 2. While the number of data points is not very large, it is clear that the correlations between estimates from the staircase method and the PSI methods are far from perfect. This is likely caused by response slowing, since the correlation coefficients are larger in the non-slowing groups, both for Experiment 1 and 2. In these groups, the adjusted PSI method produces SSRT estimates more in line with the staircase method than the PSI marginal method does. It is an open question to which degree the correlation-diminishing effects of response slowing are stemming from the PSI methods’ SSRT estimates or the staircase method’s estimates. Both the PSI methods and the staircase method might contribute to this deviation of estimates: the PSI marginal method does not take into account response slowing, and the adjusted PSI method’s estimate of Go-RTs might be suboptimal, while the staircase method might just as well not be able to deal effectively with Go-RT slowing. Future research might elucidate the source of these incongruencies in SSRT estimates.

While the method of optimal RT estimation is of technical relevance, there is also the more theoretical question of whether Go-response slowing invalidates the Stop-signal and related tasks. Indeed, it has often been pointed out that the independent horse race model underlying the analysis of behavior in this task assumes independence between the Go-process driving the Go-response and the Stop-process responsible for inhibiting this response. Since Go-response slowing in this task is generally related to the presence and prevalence of Stop-trials [15], this might call into question the validity of Stop-signal tasks that provoke considerable response slowing. We think these tasks are nevertheless important precisely because of this slowing.

While the mathematical independent horse race model understands the Go- and Stop-process as independent, it has been noted that pure independence can be paradoxical when addressing the instantiation of response stopping in the brain [16]. After all, the Stop-process must exert some form of influence over the Go-process in order to prevent its execution. Interestingly, Boucher et al. [17] showed that effects explained by the independent horse race model can be captured in an interactive horse race model where Go- and Stop-process do interact in a delayed and pronounced manner. We think that when discarding Go-response slowing simply as a nuisance when estimating SSRT, one is discarding a property inherent in the very neural inhibition system that is being studied. In this point we agree with other authors who have made Go-response slowing in Stop-signal tasks an explicit subject of experimental investigation [15, 18–20]. In this area of research, response slowing in the context of Stop-signals has often been taken as an indicator of proactive inhibition, and some results suggest that the neural processes involved in reactive response inhibition are also partly involved in proactive inhibition and can be indexed by response slowing [19, 21]. Indeed, quantifying not just reactive but also proactive inhibition in the same task has been useful in analyzing neural data and providing a more complete picture of the inhibition system [21, 22].

This is not to say, of course, that response slowing cannot be an obstacle to correct estimation of SSRT, as has been shown previously [3]. If precise estimation of only SSRT is paramount, experimental methods of preventing slowing can be crucial, such as giving participants feedback on their RTs. However, we think that in order to investigate the entire response inhibition system, Go-RT slowing must also be accounted for, which necessitates methods of SSRT estimation in the presence of slowed Go-responses.

A criticism that could be levelled against our behavioral experiments is that we used a non-traditional Stop-task, namely the Stop-change task, and included different conditions, which simulation studies usually do not. However, as described earlier, the processes involved in changing a response to another one have been found to partly overlap those of outright stopping a response [9–12]. Furthermore, while simulation studies often do not acknowledge different experimental conditions for lack of a clear way of simulating them, many behavioral experiments do include multiple conditions. Since this can increase the amount of response slowing per Stop-trial, it is important to consider the influence this has on estimating SSRTs.

We have presented behavioral as well as simulation data showing that the PSI marginal method and even the traditional staircase method in the Stop-signal task have problems accommodating excessive Go-response slowing. Consequently, we proposed an extension of the PSI method that accommodates this often observed behavior. Our results show this PSI adjusted method to be an advantageous alternative to previous methods of SSD choice especially when the number of Stop-trials that can be presented is limited.

## Supporting information

S1 Fig Illustration of the horse race model. The relationship between Go-signal, Stop-signal, SSD, SSRT and the Go-RT distribution in the Stop-signal task.
(TIF)

S2 Fig Initial- and progressive slowing of Go-RTs. Linear regression of Go-RTs on trial number throughout the PSI method’s task blocks were performed, separately for Experiment 1 (left) and Experiment 2 (right). In order to split participants into a slowed and non-slowed group, regression coefficients (progressive slowing) and intercepts (initial slowing) per participant were used as x- and y-coordinates, respectively. Then, a separating line along the main diagonal was found that splits the participants of each experiment into roughly two equally sized groups. The upper-right group is referred to as “slowing”, wheres the lower-left is referred to as “non-slowing”. The example participants from Figs 1 and 9 are shown in red.
(TIF)

S3 Fig Relationship between staircase- and PSI marginal method’s SSRT estimates in Experiment 1. The two methods’ SSRT estimates are plotted against each other, separately for slowed and non-slowed subjects. SSRTs were averaged over the four different experimental conditions. The two groups were determined as described for experiment 1, but based on the intercept and slope parameters averaged over the staircase- and PSI method’s run. Correlation coefficients are displayed, but due to the reduced sample size were non-significant.
(TIFF)

S4 Fig Different method’s performance for different degrees of response slowing. Performance of the four different methods (purple–staircase, mean method; blue–staircase, integration method; green–PSI marginal method; red–PSI adjusted method) under different degrees of response slowing.
(TIF)

S5 Fig Relationship between staircase- and PSI adjusted method’s SSRT estimates in Experiment 2. As in S3 Fig, but for experiment 2. Correlation coefficients were again non-significant due to small sample size.
(TIFF)

S6 Fig Accuracy of Go-RT prediction for different trial numbers. Accuracy of RT prediction is visualized as mean residual of predicted Go-RT (in ms; left column) and standard deviaiton of residuals (right column), for slowed (top row) and non-slowed subjects (bottom row) separately, for different trial window sizes.
(TIFF)

S1 Table Summary of behavioral performance in Experiment 1. Behavioral performance in the staircase- and PSI marginal method’s block of Experiment 1. The side (left, right) is the side on which the reaction had to be changed in the event of a Change-signal. The Go-trials of the no foreknowledge- and certain go conditions did not contain the side factor, and thus do not distinguish between left and right. CSRTs are the Change-signal task equivalent of SSRTs. For the staircase block the CSRTs were computed using the integration method, for the PSI marginal block the expected parameter corresponding to the critical SSD was derived from the ultimate posterior distribution and subtracted from the mean Go-RT. CIEs are the Change-signal task equivalent of the Stop interference effect (SIE) and are computed by subtracting a hand’s regular Go-RT from that hand’s RT when the other hand has to be inhibited.
(DOCX)

S2 Table Summary of behavioral performance in Experiment 2. (DOCX)
