# Supplementary figures and images for: A reaction-time adjusted PSI method for estimating performance in the stop-signal task
Source: PLoS One. 2018 Dec 31;13(12):e0210065. doi: 10.1371/journal.pone.0210065 (PMC6312320; doi:10.1371/journal.pone.0210065)

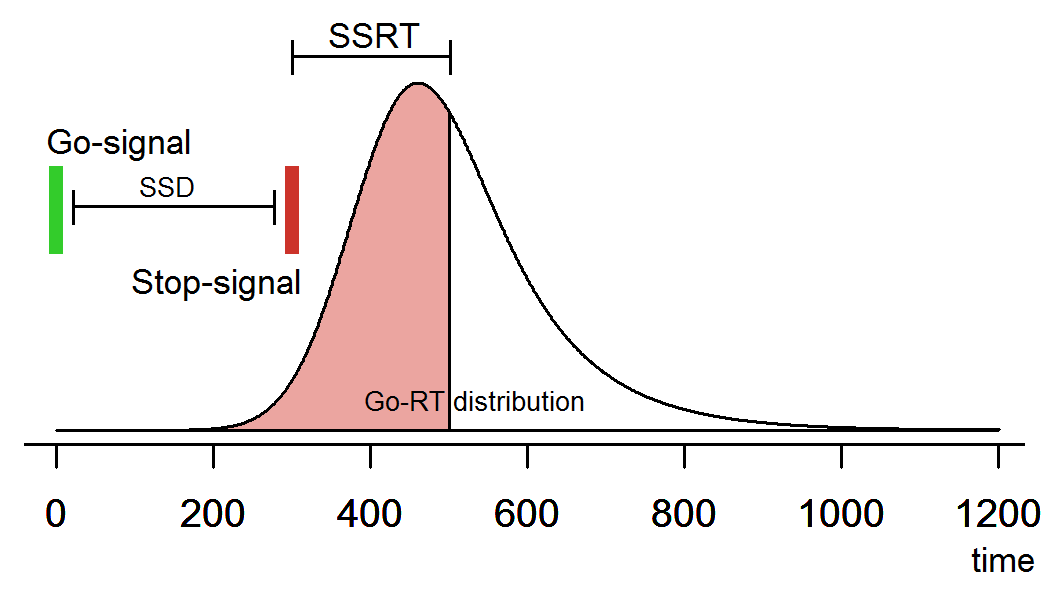

Supplement: S1 Fig — The relationship between Go-signal, Stop-signal, SSD, SSRT and the Go-RT distribution in the Stop-signal task. (TIF) [file pone.0210065.s001.tif]

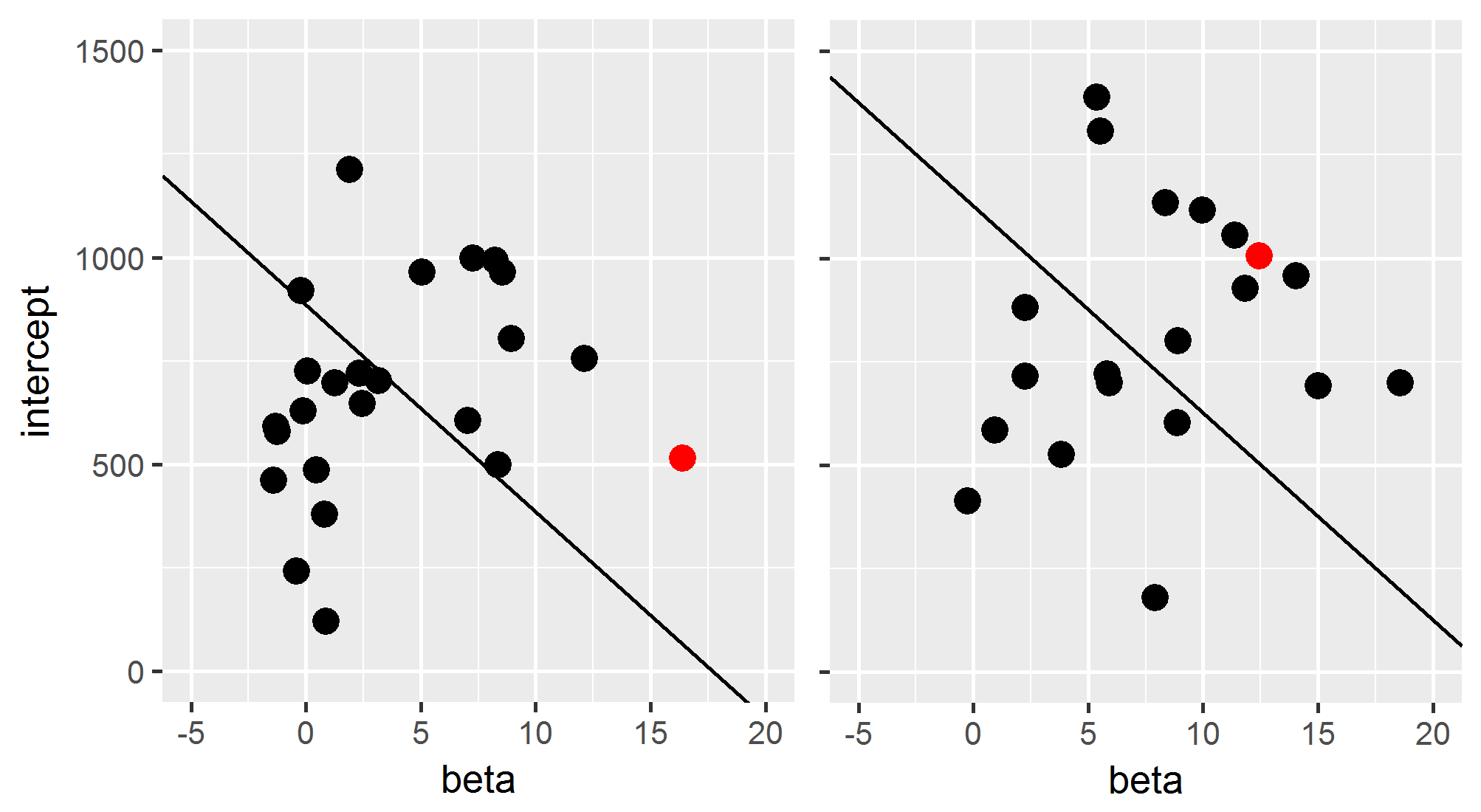

Supplement: S2 Fig — Linear regression of Go-RTs on trial number throughout the PSI method’s task blocks were performed, separately for Experiment 1 (left) and Experiment 2 (right). In order to split participants into a slowed and non-slowed group, regression coefficients (progressive slowing) and intercepts (initial slowing) per participant were used as x- and y-coordinates, respectively. Then, a separating line along the main diagonal was found that splits the participants of each experiment into roughly two equally sized groups. The upper-right group is referred to as “slowing”, wheres the lower-left is referred to as “non-slowing”. The example participants from Figs 1 and 9 are shown in red. (TIF) [file pone.0210065.s002.tif]

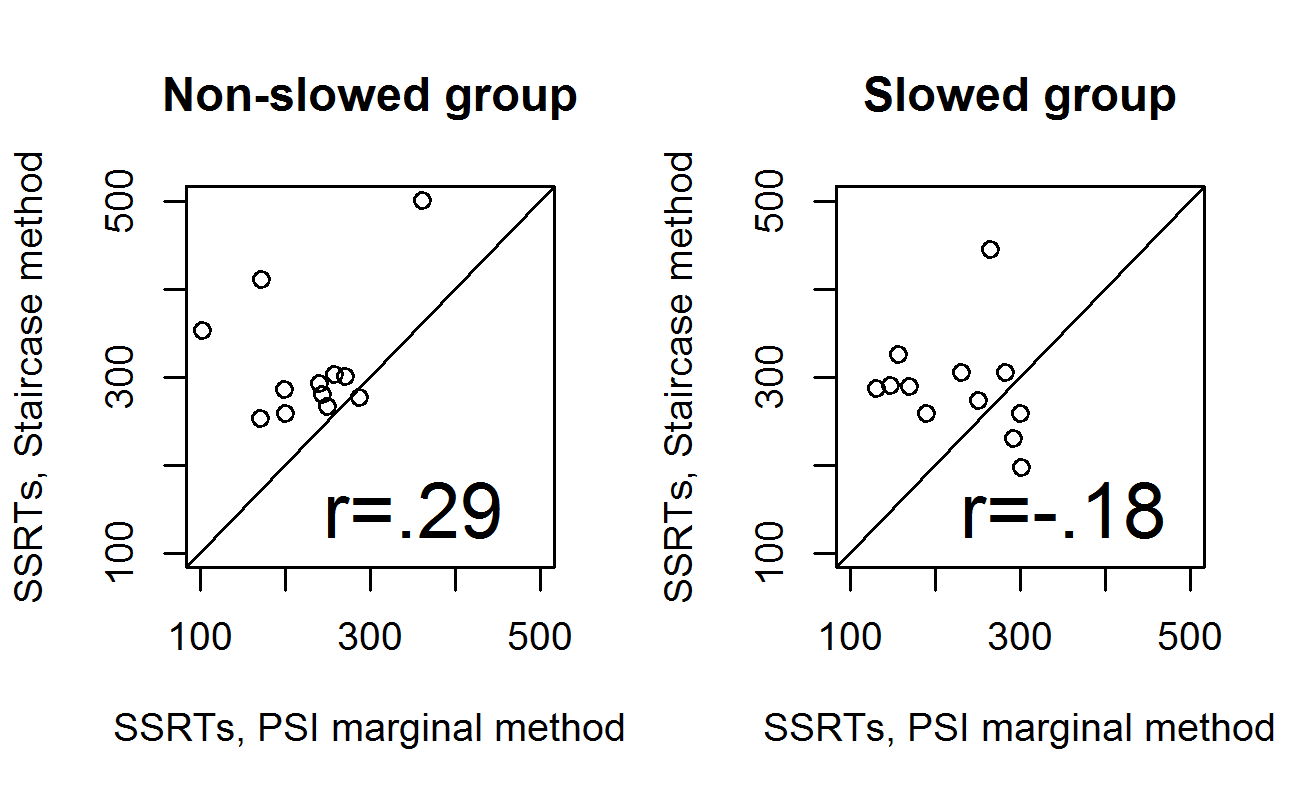

Supplement: S3 Fig — The two methods’ SSRT estimates are plotted against each other, separately for slowed and non-slowed subjects. SSRTs were averaged over the four different experimental conditions. The two groups were determined as described for experiment 1, but based on the intercept and slope parameters averaged over the staircase- and PSI method’s run. Correlation coefficients are displayed, but due to the reduced sample size were non-significant. (TIFF) [file pone.0210065.s003.tiff]

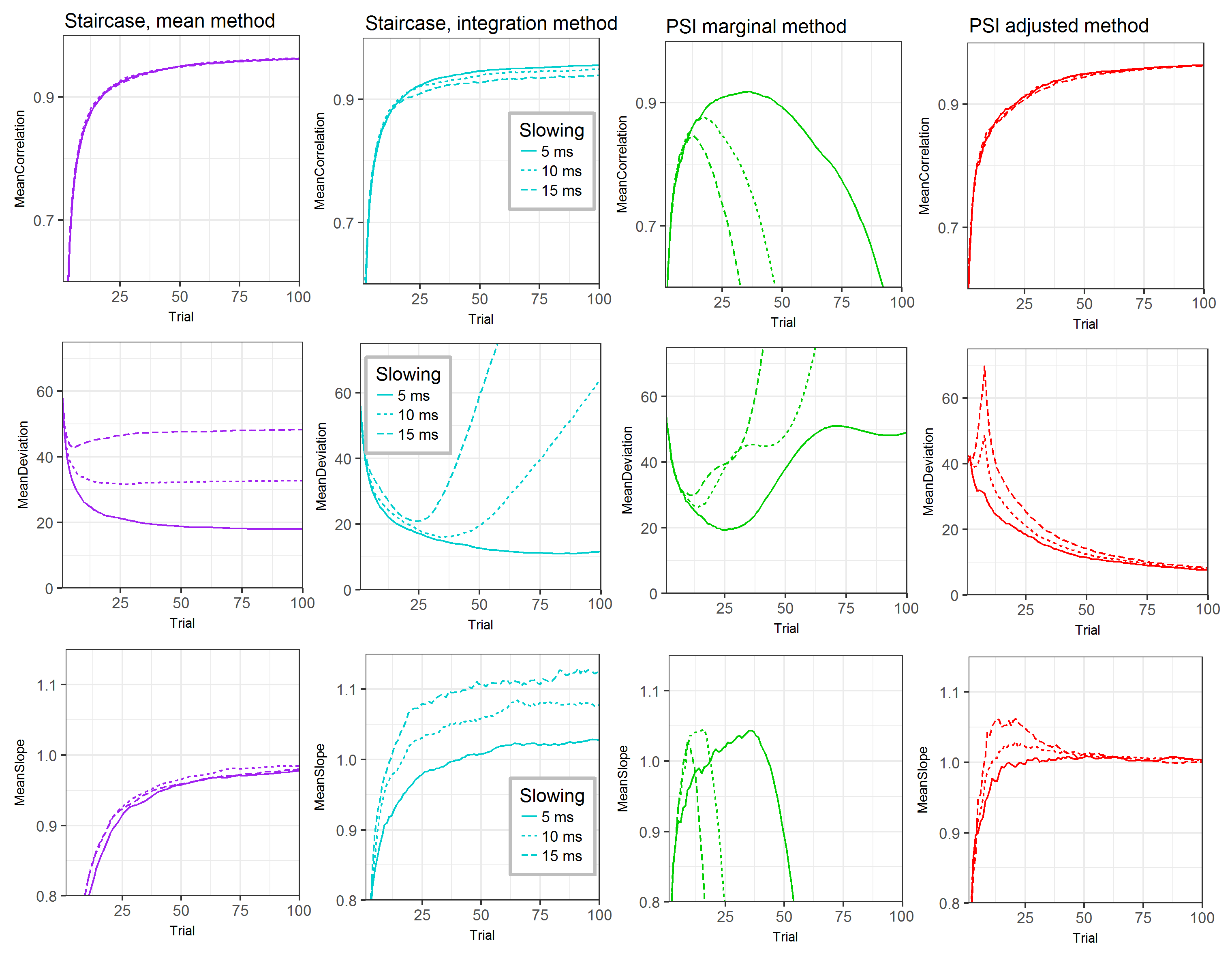

Supplement: S4 Fig — Performance of the four different methods (purple–staircase, mean method; blue–staircase, integration method; green–PSI marginal method; red–PSI adjusted method) under different degrees of response slowing. (TIF) [file pone.0210065.s004.tif]

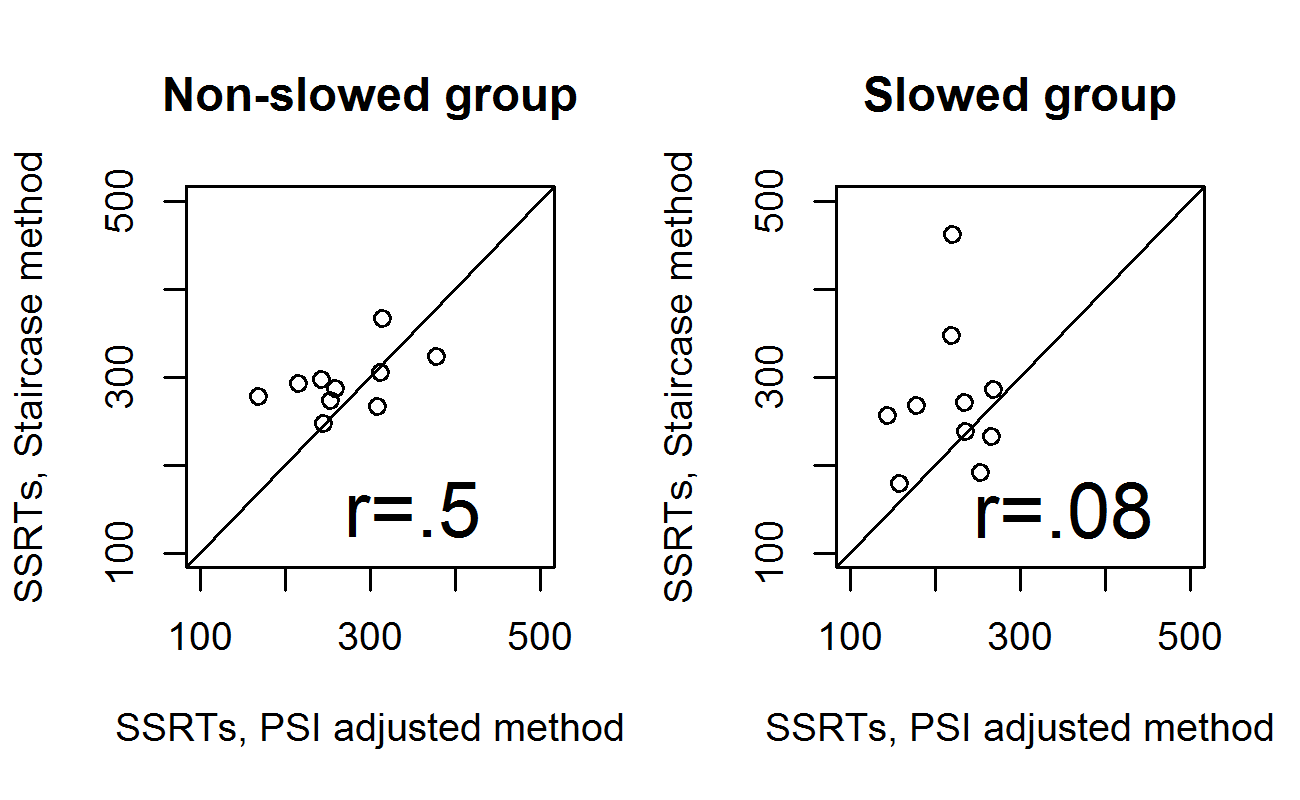

Supplement: S5 Fig — As in S3 Fig, but for experiment 2. Correlation coefficients were again non-significant due to small sample size. (TIFF) [file pone.0210065.s005.tiff]

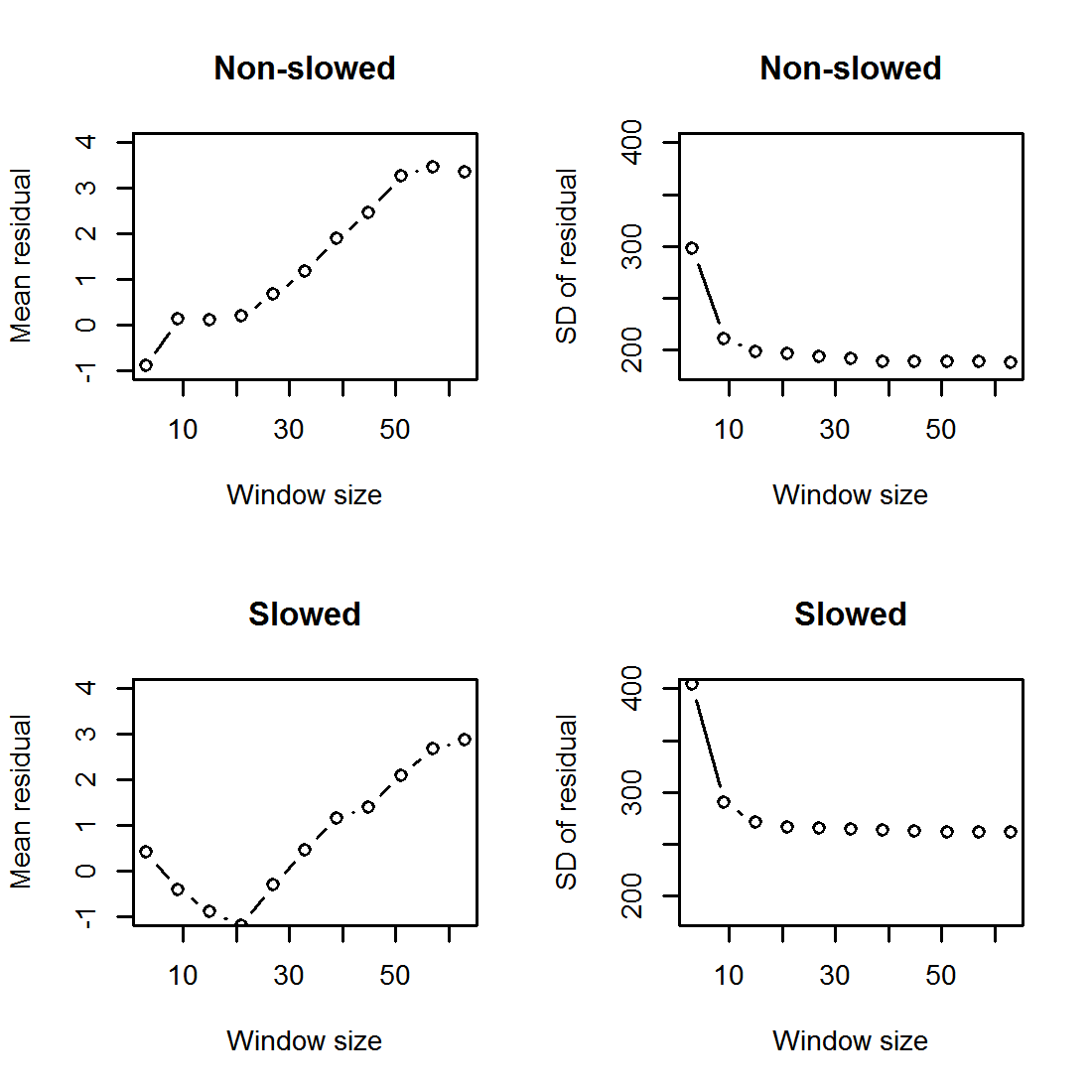

Supplement: S6 Fig — Accuracy of RT prediction is visualized as mean residual of predicted Go-RT (in ms; left column) and standard deviaiton of residuals (right column), for slowed (top row) and non-slowed subjects (bottom row) separately, for different trial window sizes. (TIFF) [file pone.0210065.s006.tiff]
