# Supplementary material for: A reaction-time adjusted PSI method for estimating performance in the stop-signal task
Source: PLoS One. 2018 Dec 31;13(12):e0210065. doi: 10.1371/journal.pone.0210065 (PMC6312320; doi:10.1371/journal.pone.0210065)
Supplement: S1 Table — Behavioral performance in the staircase- and PSI marginal method’s block of Experiment 1. The side (left, right) is the side on which the reaction had to be changed in the event of a Change-signal. The Go-trials of the no foreknowledge- and certain go conditions did not contain the side factor, and thus do not distinguish between left and right. CSRTs are the Change-signal task equivalent of SSRTs. For the staircase block the CSRTs were computed using the integration method, for the PSI marginal block the expected parameter corresponding to the critical SSD was derived from the ultimate posterior distribution and subtracted from the mean Go-RT. CIEs are the Change-signal task equivalent of the Stop interference effect (SIE) and are computed by subtracting a hand’s regular Go-RT from that hand’s RT when the other hand has to be inhibited. (DOCX) [file pone.0210065.s007.docx]

Table S1

|  | Behavioral measures, staircase block | | | | | | | | | | | | | | | | | | | | |
| --- | --- | --- | --- | --- | --- | --- | --- | --- | --- | --- | --- | --- | --- | --- | --- | --- | --- | --- | --- | --- | --- |
| Foreknowledge condition | Go-RTs | | | | |  | | | CSRTs | | | | |  | | | CIEs | | | | |
|  | *M* | |  | *SD* | | |  | *M* | | |  | *SD* | | |  | *M* | | |  | *SD* | |
|  | left | right |  | left | right | |  | left | | right |  | left | right | |  | left | | right |  | left | right |
| No foreknowledge | 738 | |  | 316 | | |  | 322 | | 319 |  | 121 | 100 | |  | 194 | | 145 |  | 269 | 169 |
| Foreknowledge | 745 | 744 |  | 299 | 302 | |  | 269 | | 298 |  | 76 | 78 | |  | 115 | | 47 |  | 181 | 129 |
| Certain Go | 284 | |  | 149 | | |  |  | |  |  |  |  | |  |  | |  |  |  |  |

|  | Behavioral measures, PSI marginal block | | | | | | | | | | | | | | | | | | | | |
| --- | --- | --- | --- | --- | --- | --- | --- | --- | --- | --- | --- | --- | --- | --- | --- | --- | --- | --- | --- | --- | --- |
| Foreknowledge condition | Go-RTs | | | | |  | | | CSRTs | | | | |  | | | CIEs | | | | |
|  | *M* | |  | *SD* | | |  | *M* | | |  | *SD* | | |  | *M* | | |  | *SD* | |
|  | left | right |  | left | right | |  | left | | right |  | left | right | |  | left | | right |  | left | right |
| No foreknowledge | 802 | |  | 347 | | |  | 244 | | 216 |  | 84 | 75 | |  | 211 | | 208 |  | 150 | 129 |
| Foreknowledge | 827 | 814 |  | 345 | 339 | |  | 249 | | 202 |  | 79 | 92 | |  | 116 | | 127 |  | 116 | 146 |
| Certain Go | 268 | |  | 119 | | |  |  | |  |  |  |  | |  |  | |  |  |  |  |
