# Supplementary material for: A reaction-time adjusted PSI method for estimating performance in the stop-signal task
Source: PLoS One. 2018 Dec 31;13(12):e0210065. doi: 10.1371/journal.pone.0210065 (PMC6312320; doi:10.1371/journal.pone.0210065)
Supplement: S2 Table — (DOCX) [file pone.0210065.s008.docx]

Table S2

|  | Behavioral measures, staircase block | | | | | | | | | | | | | | | | | | | | |
| --- | --- | --- | --- | --- | --- | --- | --- | --- | --- | --- | --- | --- | --- | --- | --- | --- | --- | --- | --- | --- | --- |
| Foreknowledge condition | Go-RTs | | | | |  | | | CSRTs | | | | |  | | | CIEs | | | | |
|  | *M* | |  | *SD* | | |  | *M* | | |  | *SD* | | |  | *M* | | |  | *SD* | |
|  | left | right |  | left | right | |  | left | | right |  | left | right | |  | left | | right |  | left | right |
| No foreknowledge | 892 | |  | 308 | | |  | 277 | | 284 |  | 89 | 66 | |  | 145 | | 91 |  | 210 | 167 |
| Foreknowledge | 882 | 891 |  | 276 | 297 | |  | 276 | | 298 |  | 84 | 69 | |  | 43 | | 14 |  | 138 | 147 |
| Certain Go | 241 | |  | 158 | | |  |  | |  |  |  |  | |  |  | |  |  |  |  |

|  | Behavioral measures, PSI adjusted block | | | | | | | | | | | | | | | | | | | | |
| --- | --- | --- | --- | --- | --- | --- | --- | --- | --- | --- | --- | --- | --- | --- | --- | --- | --- | --- | --- | --- | --- |
| Foreknowledge condition | Go-RTs | | | | |  | | | CSRTs | | | | |  | | | CIEs | | | | |
|  | *M* | |  | *SD* | | |  | *M* | | |  | *SD* | | |  | *M* | | |  | *SD* | |
|  | left | right |  | left | right | |  | left | | right |  | left | right | |  | left | | right |  | left | right |
| No foreknowledge | 1086 | |  | 389 | | |  | 255 | | 253 |  | 86 | 83 | |  | 238 | | 209 |  | 141 | 149 |
| Foreknowledge | 1087 | 1088 |  | 382 | 374 | |  | 246 | | 220 |  | 77 | 67 | |  | 127 | | 127 |  | 124 | 120 |
| Certain Go | 224 | |  | 161 | | |  |  | |  |  |  |  | |  |  | |  |  |  |  |
